# Lifelong impact of ENPP1 Deficiency and the early onset form of ABCC6 Deficiency from patient or caregiver perspective

**DOI:** 10.1371/journal.pone.0270632

**Published:** 2022-07-27

**Authors:** Christine O’Brien, Gus Khursigara, Pedro Huertas, Beth Leiro, Liz Molloy, Catherine Nester

**Affiliations:** 1 GACI Global, Argyle, Texas, United States of America; 2 Inozyme Pharma Inc, Boston, Massachusetts, United States of America; 3 Mirror Neuron Partners LLC and Harvard–MIT Program in Health Sciences and Technology, Boston, Massachusetts, United States of America; University of Basilicata, ITALY

## Abstract

The ectonucleotide pyrophosphatase/phosphodiesterase family member 1 (ENPP1) and ATP-binding cassette subfamily C member 6 (ABCC6) proteins play a prominent role in inhibiting ectopic calcification and arterial stenosis. Patients with ENPP1 Deficiency or infant onset ABCC6 Deficiency often present with pathological calcification, narrowed blood vessels, multiorgan dysfunction and high infant mortality. The heterogenous presentation and progression is well documented. Our objective was to characterize how these morbidities lead to burden of illness and poor quality of life across ages from the patient/caregiver perspective. Patients/caregivers were interviewed via phone using Institutional Review Board–approved questionnaires. Patient-reported outcomes were collected via validated instruments. Thirty-one caregivers and 7 patients participated: infant onset ABCC6 Deficiency, n = 6 (infants/children); ENPP1 Deficiency, n = 32 (13 infants, 12 children, 7 adults). ENPP1 and ABCC6-deficient children aged <8 years and aged 8–18 years reported poor school functioning (0.69 vs 0.72 effect size, respectively) and poor physical health (0.88 vs 1, respectively). In the total ENPP1 cohort, 72% (23/32) reported bone/joint pain and/or mobility/fatigue issues. Three of seven ENPP1-deficient adults reported moderate to severe pain (>4), as measured by the Brief Pain Inventory (BPI), that interfered with daily activities despite pain medication. Top reported burdens for caregivers of infants with ABCC6/ENPP1 Deficiencies included heart-related issues and hospitalizations. Treatment/medications, and hearing loss were the highest burdens reported by caregivers/families of the pediatric ENPP1 Deficiency cohort, whereas adults reported bone/joint pain and mobility impairment as the greatest burdens. Individuals with ENPP1 Deficiency or infant onset ABCC6 Deficiency experience lifelong morbidity causing substantial physical and emotional burden to patients/caregivers.

## Introduction

Ectonucleotide pyrophosphatase/phosphodiesterase family member 1 (ENPP1) Deficiency is a systemic and progressive disorder associated with early mortality, vascular calcification, and skeletal complications [1,2]. Deficiency in ENPP1 enzyme function results in low levels of pyrophosphate (PPi) and adenosine monophosphate (AMP) leading to calcification of arteries, arterial stenoses, cardiac complications, and rickets/osteomalacia [1,3]. The condition is inherited as a recessive trait with a frequency of approximately 1:200,000 pregnancies [2].

ENPP1 Deficiency presents and manifests across all age groups [3]. Infants with ENPP1 Deficiency present with generalized arterial calcification of infancy (GACI) (also described as idiopathic infantile arterial calcification), characterized by low PPi levels, calcification of multiple arteries, cardiac complications (ie, reduced ejection fraction, myocardial infarction, severe hypertension, heart valve defects), and cardiac failure [3–5]. Mortality is the highest during the infantile phase (ranging from 41% to 70% of infants with ENPP1 Deficiency) and occurs predominantly in the first 6 months of life [3,4]. Patients can also experience seizure, stroke, gastrointestinal complications, and renal dysfunction [3,4,6]. In its pediatric form, patients who survive infancy or newly diagnosed children without a GACI phenotype, go on to develop fibroblast growth factor 23-mediated autosomal recessive hypophosphatemic rickets type 2 (ARHR2) with continued risk of calcification, cardiovascular complications, and hearing deficits [2,3,7]. Long-term morbidities include severe bone pain, enthesopathies, and increased risk of bone fracture [3,8]. The adult form of ENPP1 Deficiency has similar characteristics to the pediatric form, including skeletal deformities, osteomalacia, osteoarthritis, and interosseous membrane ossification [2,9].

The prominent role of transmembrane protein ENPP1 is to hydrolyze adenosine triphosphate (ATP) to PPi and AMP, potent inhibitors of ectopic calcification and intimal proliferation of arteries, respectively [10–13]. It is proposed that the ABCC6 protein (ATP-binding cassette subfamily C member 6) facilitates the release of ATP from intracellular hepatocytes into the extracellular space where it serves as substrate for ENPP1, thus linking a common PPi pathway between ENPP1 and ABCC6 [12,13].

Similar to patients with ENPP1 Deficiency, patients with a deficiency in ABCC6 also have low levels of plasma PPi [13]. This deficiency commonly presents in the adult phase, diagnosed as pseudoxanthoma elasticum (PXE), affecting approximately 1/25,000 to 1/ 50,000 people worldwide [14]. PXE manifests around the second decade of life with a pattern of progressive calcification of elastic fibers in the skin and Bruch’s membrane of the eye (angioid streaks), retinal bleeding, vision loss, and peripheral vascular disease [15]. However, in rare cases, a deficiency in ABCC6 can present in infancy (infant onset) with a GACI phenotype indistinguishable from that of infants with ENPP1 Deficiency, accounting for 9% to 15% of infants with GACI [3,4,16]. Despite the significant clinical overlap, the risk of mortality is lower compared to ENPP1 Deficiency (~15% vs. ~50%) and there does not appear to be a risk for hypophosphatemic rickets or hearing loss [3].

Due to the overlapping pathophysiology and clinical presentation in infants with ENPP1 Deficiency and infants that are deficient in ABCC6, treatment for both genetic deficiencies are similar and supportive in nature. In infants, antihypertensives, extracorporeal membrane oxygenation, vasopressors, and intubation are used to manage cardiovascular complications. In children and adults with ENPP1 Deficiency, oral phosphate, calcitriol, and surgical intervention are used to manage skeletal abnormalities. Inorganic PPi analogues such as bisphosphonates are used try to reduce vascular calcification and improve survival; however, a recent study showed no significant survival benefit with bisphosphonate treatment when started after the first week of life [3].

While research continues to broaden our understanding of the clinical course of both ENPP1 and ABCC6 Deficiencies, there is a lack of understanding surrounding the impact of these multisystem diseases on the patient and caregiver. To date, most research in ENPP1 and ABCC6 Deficiencies has focused on the genetics, biochemistry, and clinical manifestations of the diseases. Additional research is needed to understand more clearly the patient experience as it relates to the burden of illness, and the firsthand impact on the lives of affected patients and their families. Here we report findings from a non-interventional, multinational survey conducted to characterize the burden of illness from the patient and caregiver perspective. Infants with ENPP1 Deficiency and infant onset ABCC6 Deficiency have an indistinguishable presentation and a similar course of supportive care. PXE has a distinct clinical course thus our study included both infant onset ABCC6 Deficiency and ENPP1 Deficiency but does not address adults diagnosed with PXE.

## Materials and methods

### Study design and methodology

This was a cross-sectional study (NCT04372446) conducted in patients/caregivers of patients with ENPP1 Deficiency or infant onset ABCC6 Deficiency and was sponsored by Inozyme Pharma, Inc. Eligible study participants completed the patient-reported outcome (PRO) tools followed by a 40-minute follow-up interview conducted by a trained interviewer to record the burden of illness. The burdens were captured at any time point over the course of the patient’s lifetime. The study design and questions were developed as a collaboration between Engage Health, Inc., the GACI Global patient advocacy group, and researchers from Inozyme Pharma, Inc. Representatives from Engage Health, Inc. conducted the patient interviews. The responses were entered into a proprietary US Health Insurance Portability and Accountability Act (HIPAA)-compliant study module from Engage Health, Inc. If the participant consented, the interview was recorded and stored on HIPAA-compliant servers. Statistical analysis was performed by Engage Health and Inozyme Pharma based on a pre-determined statistical analysis plan. Central Institutional Review Board approval was obtained prior to the initiation of the study. All participants provided electronic informed consent through a secure site.

### Study population

Participants were recruited from Germany, France, Italy, the United Kingdom, Ireland, Canada, Australia, Denmark, and the United States. Patients were recruited via various sources, including recruitment letter to members of the GACI Global patient advocacy group, social media, and the proprietary EnCompass^®^ database (Engage Health, Inc.), of which patients in the database have opted in to be notified of research opportunities. Recruitment materials describing the study and eligibility requirements were provided in supplementary material as well as via a web page describing the study. Thirty-nine patients/caregivers responded and 38 were accepted for the study.

All participants had to meet the following criteria: 1) ≥18 years old with proof of diagnosis of ENPP1 Deficiency, or 2) caregiver of an infant or pediatric patient <18 years old (alive or deceased) with proof of a diagnosis of ENPP1 Deficiency, or 3) caregiver of a patient diagnosed with infant onset ABCC6 Deficiency that presented in infancy (alive or deceased). Diagnosis could be confirmed with genetic report, letter from the treating healthcare professional, notes from the medical record, or school documentation showing accommodation of disease. Participants also had to be able to participate in the survey via interview in German, French, or English. For the patient-reported outcomes portion of the study, a completed online assent form was required for participants 12 to 18 years of age.

### Patient-reported outcomes

The Ages & Stages Questionnaires, Third Edition (ASQ-3) is a developmental screening tool for children aged 1 month to 66 months used in this study for participants from infancy to 36 months of age. The ASQ-3 provides age-specific questionnaires at 2-month intervals for ages 2 months through 24 months, and at 3-month intervals for ages 24 to 36 months. These surveys, completed by caregivers, assess the patient’s communication, gross and fine motor skills, problem-solving skills, and personal/social skills. The ASQ-3 identifies whether children are on track with development, require monitoring, or require further assessment by a healthcare professional. Permission to use the ASQ-3 was obtained through the Paul H. Brookes Publishing Co., Inc.

Pediatric Quality of Life Inventory (PedsQL) is a modular instrument for measuring health-related quality of life (HRQoL) beginning at age 2 through adulthood with parent proxy used for the youngest children and self-report combined with parent proxy for older children. Questionnaires are age specific and designed to measure function in the core dimensions of health as outlined by the World Health Organization, as well as school functioning. Scores are generated for each of the four scales: Physical Functioning, Emotional Functioning, Social Functioning, and School Functioning. In addition, summary scores are generated for Physical and Psychosocial Health as well as a Total Summary score. Permission to use the PedsQL tool was obtained through Mapi Research Trust.

Patient-Reported Outcomes Measurement Information System (PROMIS) is a set of measures that evaluates physical, mental, and social health in adults and children. For this study, participants answered questions on a Physical Function 12-item custom short form, which assesses physical difficulty of performing tasks on a scale of 1 (unable to perform task) to 5 (able to perform task without difficulty). The items on the custom short form were informed by a review of the medical literature, the natural history study of GACI, and input from families affected by GACI or ARHR2. Permission to use the PROMIS Custom Short Form for this study was obtained from the Department of Medical Social Sciences at Northwestern University Feinberg School of Medicine. The degree of impairment of physical function was measured based on patient classification of clinical severity in adults with rheumatic diseases [17], as follows: >65, no impairment in physical function; 45–65, mild impairment in physical function; 35–45, moderate impairment in physical function; <35, severe impairment in physical function.

To assess pain, all adult patients and participants aged 12 to 18 years completed the Brief Pain Inventory (BPI) questionnaire. Caregivers completed the survey for participants aged 3 to 12 years. Permission to use the BPI Short Form (BPI-SF) was provided by The University of Texas MD Anderson Cancer Center. Two separate domains are measured by the BPI—pain intensity (severity) and the impact of pain on functioning (interference). Respondents score their pain at its worst, least, average, and current levels. In addition, a composite of the four pain items (a mean severity score) is calculated. Each item is scored from 0 (no pain) to 10 (worst pain you can imagine). Additional data collected with the BPI tool is the measure of how much pain has interfered with seven activities of daily living. Pain interference is calculated as the mean of the seven interference items. Each item is scored from 0 (does not interfere) to 10 (completely interferes). Interventions used to treat pain and the response rates were also collected. For those aged 12 to 18 years, an assent form was completed prior to taking the survey.

### Participant interviews

Trained interviewers conducted a 40- to 60-minute phone interview in the patient or caregiver’s native language or in English (language was selected by the participant). To understand the specific burdens of the disorder from the patient/caregiver perspective, respondents were asked to select all symptoms the patient has experienced as a result of their disease from a list of symptoms that was prepared after review of the medical literature, the natural history study of GACI, and input from families affected by GACI orARHR2. From the same list, participants were then asked to identify which symptoms had the most impact on them or their child. Additionally, participants were asked to list burdens with the most, second-most, and third-most impact to them in an unaided manner (ie, free form), and the weighted summary score was calculated. If the participant did not wish to answer any question in the study, they were given the option to abstain. All the respondents’ answers were considered Anonymous Data.

### Data collection, quality control, and statistical analysis

For data collection and analysis, participants were identified by code only, and quality control was performed by independent coders. For quality control, participants were contacted by Engage Health, Inc. if any data needed clarification. Following quality control, all study data were downloaded by Engage Health, Inc. and made pseudonymous to remove identifying information and each participant was assigned a unique identifier. The identification numbers were stored separately from the anonymized data. The study data were locked following this, and the pseudonymous answers of participants were then combined for analysis. The data for each study subgroup were analyzed and reported by Engage Health, Inc. using inductive and descriptive statistical approaches as appropriate for the data collected. For the inductive approach, information was gathered from responses in the words of participants with two independent coders using MAXQDA 2020 (VERBI Software, 2019) to analyze the comments and subtext and assigned a code for the “burden” as well as the “life impact.” If there was a disagreement between the coders, a third party independently assessed the data. For the deductive statistical approach, participants chose the top-ranked burdens from a list compiled from the medical literature.

Burden of disease assessments included physical health related to heart issues, gastrointestinal issues, bone/joint pain, joint stiffness, mobility and fatigue, growth and development, feeding issues, short stature and developmental delays, hearing loss, and renal impairment; social health: peer and family relationships; and mental health: fear of unknown, stress/anxiety. Expected life impacts included self-esteem/self-confidence, connection with others, financial burdens, time commitment, independence, and inclusion.

## Results

### A. Study population

A total of 38 respondents representing 29 families (31 caregivers, 7 patients) were enrolled across 4 cohorts—6 with infant onset ABCC6 Deficiency and 32 with ENPP1 Deficiency (Table 1). Of the 6 respondents for the ABCC6-deficient cohort, there were 5 infants, 1 adolescent, 3/6 female, 5/6 White, and all 6 were alive at enrollment. All had a diagnosis of GACI due to ABCC6 Deficiency, and the mean age at diagnosis was 0.3 years.

**Table 1:** Demographics and characteristics of the study population.

	ABCC6 Deficiency	ENPP1 Deficiency			
	Infants and children (n = 6)	All (n = 32)	Infants (0<2 years) (n = 13)	Children (2-<18 years) (n = 12)	Adults (≥18 years) (n = 7)
Age					
Mean, y	2.5	10	0.3	7.9	31.6
Median (range), y	2 (1–5 years)	5 (1–50 years)	0 (1–2 years)	7 (2–15 years)	30 (20–50 years)
Sex, n (%)					
Female	3 (50)	15 (46.9)	4 (33.3)	8 (61.5)	3 (42.9)
Male	3 (50)	17 (53.1)	9 (69.2)	4 (33.3)	4 (57.1)
Race/Ethnicity, n (%)					
White	5 (83.3)	22 (68.8)	9 (66.7)	8 (69.2)	5 (71.4)
Asian or American Indian	3 (25.0)	5 (15.6)	2 (15.3)	0 (0.0)	0
Black/African/Aboriginal/Australian	0	2 (6.3)	1 (8.3)	1 (7.7)	0
Turkish	0	2 (6.3)	0	0	2 (28.6)
White and Black African	0	0	0	0	1 (16.7)
White and Asian/American Indian	0	1 (3.1)	0	1 (7.7)	0
Vital status, n (%)					
Alive	6 (100)	21 (65.6)	3 (23)	11 (91.6)	7 (100)
Deceased	0	11 (34.3)	10 (76.9)	1 (8.3)	0
Diagnosis, n (%)					
GACI	0	21 (65.6)	13 (100)	7 (58.3)	1 (14.2)
GACI due to ABCC6	6 (100)	0	0	0	0
GACI and ARHR2	0	6 (18.8)	0 (0.0)	4 (30.1)	2 (28.6)
ARHR2	0	5 (15.6)	0 (0.0)	1 (7.7)	4 (57.1)
Age at diagnosis, y					
Mean	0.3	4.0	0	1.5	15.6
Median	0	0	0	0	12

ABCC6, ATP-binding cassette subfamily C member 6; ARHR2, autosomal recessive hypophosphatemic rickets type 2; ENPP1, ectonucleotide pyrophosphatase/phosphodiesterase family member 1; GACI, generalized arterial calcification of infancy.

For the ENPP1-deficient cohort (n = 32), 13 (41%) were infants (0–2 years), 12 (38%) were children (>2-<18 years), and 7 (22%) were adults. The median ages at diagnosis were 0, 1.5, and 15.6 years, respectively. Fifteen (47%) were female, 17 (53%) were male, and the majority were white (n = 22; 69%). Sex was not considered a factor in the statistical analysis of these data. Twenty-one were alive (66%) and 11 (34%) were deceased; 10 (31%) died before 12 months of life, 21 (66%) had a GACI diagnosis, 5 (16%) had ARHR2, and 6 (19%) had GACI and ARHR2. Eleven (34%) of the respondents had a family history of rickets/GACI.

### B. Patient-reported outcomes

**Fig 1:**
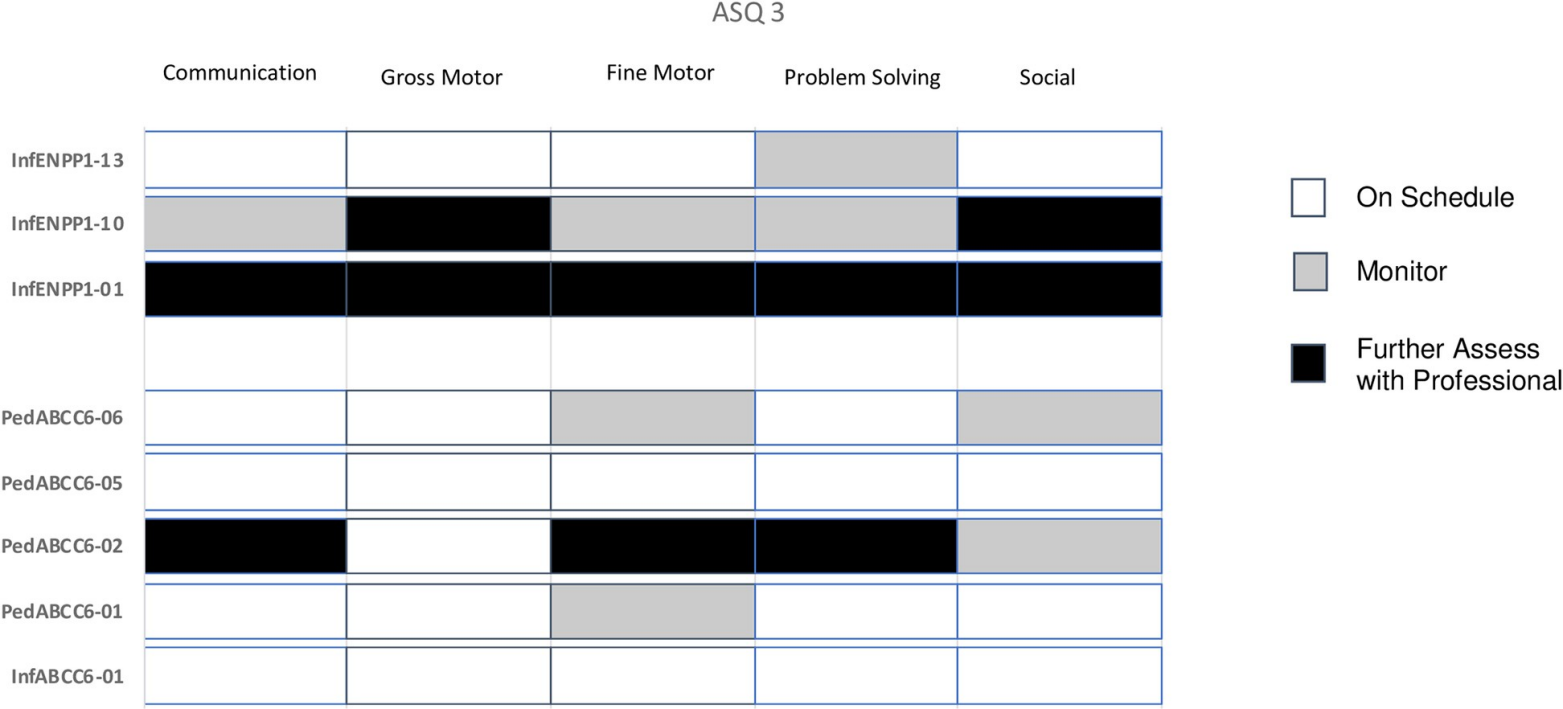
ASQ-3 instrument scores for infants/children with ENPP1 Deficiency/ infant onset ABCC6 Deficiency.

ASQ-3 was completed for 5 respondents in the infant onset ABCC6 Deficiency cohort and 3 respondents in the ENPP1 Deficiency cohort (Fig 1). Only 2 of the 5 infant onset ABCC6 Deficiency respondents showed development on schedule for all 5 domains. One of the 5 infant onset ABCC6 Deficiency respondents showed delayed development in 3 of the 5 domains, and 2 other respondents showed borderline delay requiring monitoring in fine motor skills. None of the respondents in the ENPP1 cohort demonstrated age-appropriate development in all the domains assessed by the ASQ-3. Two of the 3 respondents in the ENPP1 cohort showed delayed development in at least 2 of the 5 domains, with one respondent showing delayed development in all 5 domains. The last respondent showed delayed development in problem solving.

HRQoL was evaluated for living pediatric patients who presented with a GACI phenotype due to ENPP1 Deficiency or infant onset ABCC6 Deficiency using age specific PedsQL instruments. Eleven of the 12 children with ENPP1 Deficiency and 1 of the 6 children with infant onset ABCC6 Deficiency were evaluated using the appropriate age-based form and then stratified into two groups, <8 years or ≥8 years of age. Overall, the mean total PedsQL score was low for the study population compared with that for healthy children [18], as measured by large and moderate effect sizes (Table 2). Each domain that was measured (emotional function, social function, school function, and physical health) had size effect ranging from small to large across both age groups, which demonstrated impact on quality of life. Physical health and school function domains were impacted the most in both age groups, as reflected by large to moderate effect sizes, respectively (Table 2).

**Table 2:** PedsQL scores for children With ENPP1 Deficiency and infant onset ABCC6 Deficiency vs healthy children.

	Children aged<8y (n = 1)	Children aged <8 y (n = 6)			Children aged ≥8 y (n = 5)		
	*ABCC6 variants*	*ENPP1* variants	Healthy	Effect size^a^	*ENPP1* variants	Healthy	Effect size
Emotional function, mean (SD)	60	64.2 (12.9)	77.5 (18.3)	0.73	75 (31.2)	80.6 (17.4)	0.32
Social function, mean (SD)	40	80.8 (22.2)	82.9 (21.3)	0.10	72 (22.0)	81.2 (22)	0.28
School function, mean (SD)	20	75.5 (29.5)	80.9 (19.2)	0.28	57.2 (21.1)	76.2 (19.6)	0.72
Physical health, mean (SD)	12.5	86.5 (21.9)	89.5 (15.5)	0.19	76.6 (30)	87 (17)	0.61
Total, mean (SD)	30	78.2 (19.7)	83.7 (15.2)	0.36	71 (15.7)	82 (15.1)	0.73

ABCC6, ATP-binding cassette subfamily C member 6; ENPP1, ectonucleotide pyrophosphatase/phosphodiesterase family member 1; PedsQL, Pediatric Quality of Life Inventory.

^a^Magnitude of effect size: Ignorable (<0.2), small (0.2–0.49), moderate (0.5–0.79), large (≥0.8).

Pain assessments via BPI-SF were available for 11 children and 7 adults with ENPP1 Deficiency. For adult respondents, the mean scores for pain severity and pain interference in daily activities were 4.3 (mild to moderate) and 4.2 (moderate), respectively (Table 3) [19]. The mean BPI-SF scores in adult respondents for worst pain, least pain, average pain, and pain at the time of assessment were 4.9, 3.1, 4.3, and 4.9, respectively. Three of the 7 adults reported moderate or severe pain all of whom also reported severe interference of daily activity due to pain. Three other adults reported mild pain with moderate interference of daily activities due to pain and 1 adult did not report pain (Table 3). Location of pain is presented in Table 3, with majority occurring in hip, knee, shoulders, and neck. Pain medication was used by 5/7 adults and provided 20% to 70% relief of pain. Only 5 of the 11 respondents for children recorded pain severity scores, with 4 reporting mild pain and 1 reporting moderate pain. Two additional patients reported knee pain but had pain severity scores of zero. (Table 3).

**Table 3:** BPI scores.

Subject ID	BPI- Mean Pain Severity Score	BPI- Mean Pain Interference Score	Pain Location	Treatments	% Relief from Treatment
AdultENPP1-01	3.75	3.1	Pain in neck, L hip, L knee, neck, and low back	Paracetamol, Naproxen, Elavil, Physiotherapy, and co-codamol	50%
AdultENPP1-02	7	6.57	Right Shoulder, arm, hand, hip, foot, L hip, upper back, and head	Sinemet massage, methocarbamol	20%
AdultENPP1-03	5.25	5.71	Knees, ankles, neck, upper back, low back	Ibuprofen 600 mg	70%
AdultENPP1-04	3.75	4	Back, R hip, R knee, R ankle, neck, and both shoulders	Arthrex and Schmerzgel (pain gel)	30%
AdultENPP1-05	7.25	6.86	Neck, shoulders, elbows, knees wrists, ankles, and hips	Voltaren, Ibuprofen, and Emulgel	50%
AdultENPP1-06	3	3.14	R knee	-	-
AdultENPP1-07	0	0	None	-	-
A Mean	**4.29**	**4.20**			
PedENPP1-01	2	0	Pain in sternum, headache. Bilateral shins	Phos and Vitamin D Paracetamol, and Ibuprofen	0%
PedENPP1-02	0	0			
PedENPP1-03	0	0	Bilateral Knees	Phos Calcium, Ibuprofen and Tylenol	
PedENPP1-04	0	0	Pain in knees	Ibuprofen, Tylenol, Phosphorus, and Calcitriol	
PedENPP1-05	0	0	None	None	
PedENPP1-06	0	0	None	None	
PedENPP1-07	3.5	3.43	Bilateral anterior thighs, bilateral knees, bilateral calves, anterior and posterior	Phosphate, Calcitriol, and Panadol	
PedENPP1-09	0.75	0.57	Pain in upper back, knees, and ankles	None	
PedENPP1-10					
PedENPP1-11	1.5	1	Knees and head	Panadol	100%
PedENPP1-12	1	1.14	Anterior aspect of legs	None	
PedENPP1-13	0	0	None	None	
P mean	**0.80**	**0.56**			

BPI, Brief Pain Inventory; ENPP1, ectonucleotide pyrophosphatase/phosphodiesterase family member 1; L, left; Ped, pediatric; R, right.

BPI Interference Score: <2 non/mild; 2–5 moderate; >5–10 severe [19].

BPI Severity Score: 1–4 mild pain, 5–6 for moderate and 7–10 for severe pain.

**Fig 2:**
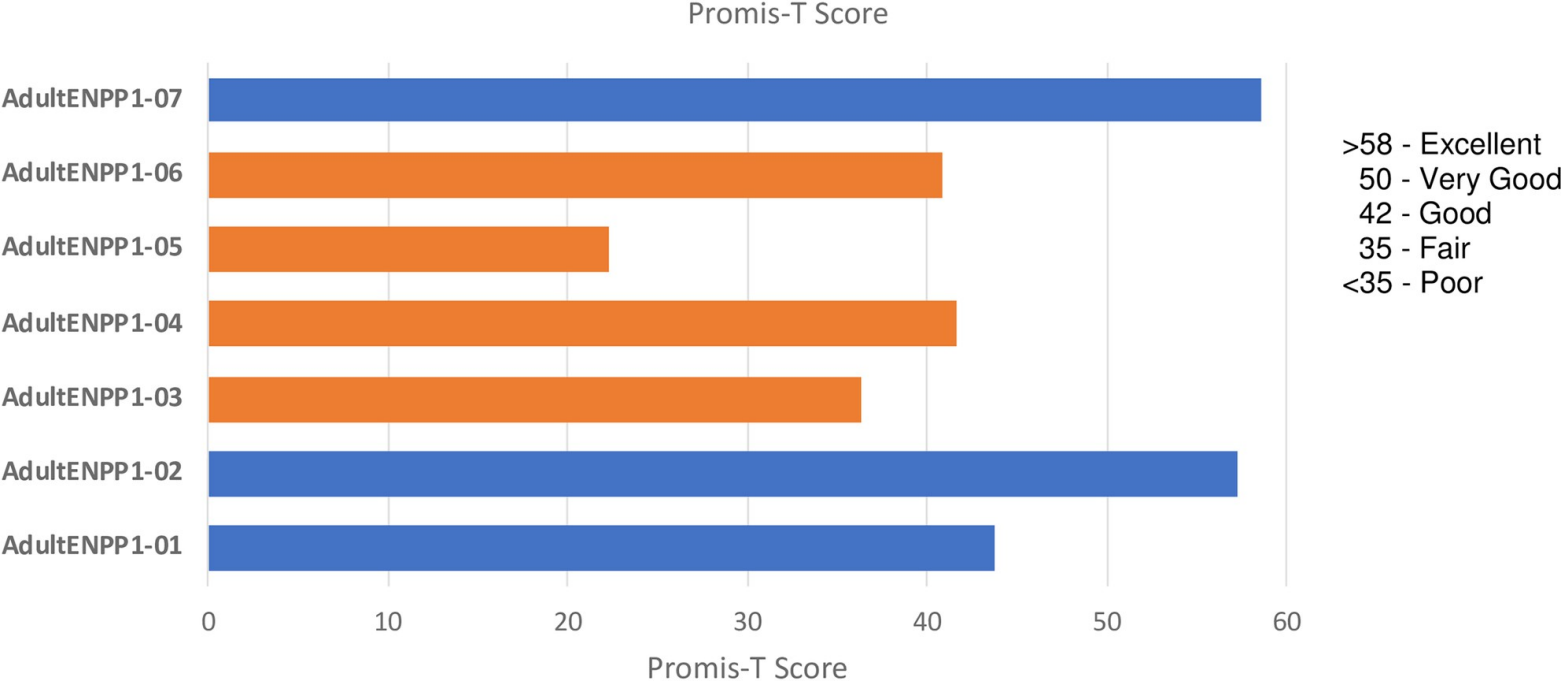
PROMIS scores for adults with ENPP1 Deficiency.

PROMIS scores are represented as T-scores, which are based on a mean of 50 and a standard deviation of 10. The PROMIS Global Physical Health T-score showed reduced physical health in adults with ENPP1 Deficiency. Mean (SD) PROMIS T-score was 42.9 (12.4), and 5/7 respondents (71%) had summary PROMIS T-scores of physical health subdomains lower than the US general population mean of 50. Four of seven self-rated their health as poor to fair (Fig 2). Four of the seven adult patients also noted bone/joint pain has worsened over time despite the use of pain medication.

### C. Symptoms related to vascular/organ complications

**Fig 3:**
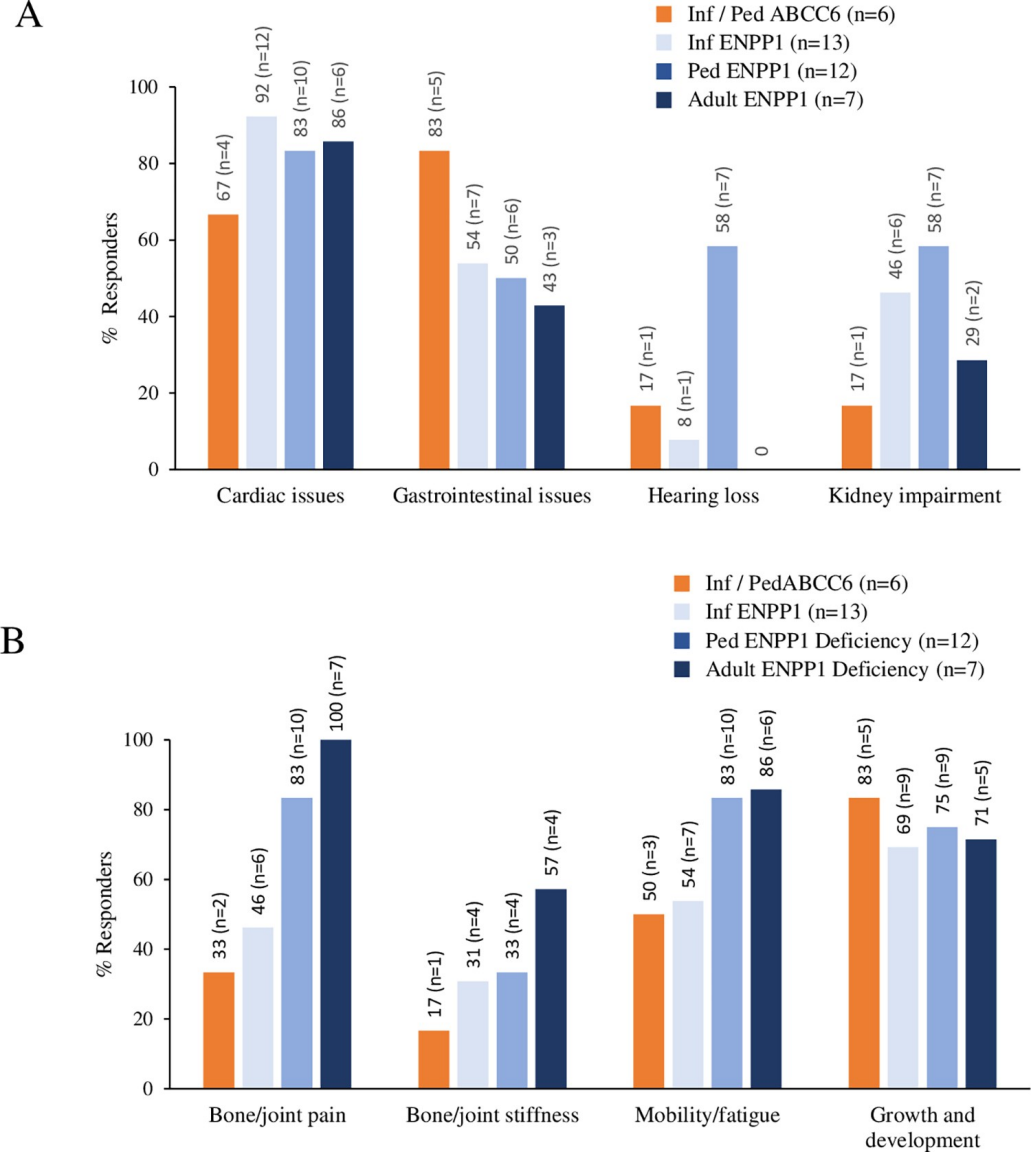
**Patient/Caregiver-reported infant onset ABCC6 Deficiency and ENPP1 Deficiency–related manifestations at all time points measured indicative of (A) organ involvement, (B) skeletal issues/impairment of mobility.** Analysis included hearing loss and cardiac, gastrointestinal, kidney, and musculoskeletal (as measured by mobility and fatigue) impairment.

The most common disease-related symptoms in infants with ABCC6 Deficiency (n = 6) were cardiac (4/6) and gastrointestinal (5/6) symptoms (Fig 3A). Cardiac issues included calcification of arteries/aorta/heart, cardiac failure, and myocardial infarction (S1 Table). All infants with cardiac involvement showed symptoms by 3 months of life, per caregiver reports. Cardiac issues improved over time in all 4 infants with ABCC6 Deficiency.

In the ENPP1-deficient cohort, cardiac, gastrointestinal, hearing, and renal symptoms were reported across all age groups (Fig 3A). Hearing loss was observed in infants and children but not in adults (Fig 3A). Overall, 41% reported symptoms across 3 or more organ systems. Cardiac involvement was reported in 28/32 patients in the ENPP1-deficient cohort, with prevalence ranging from 83% to 92% across the different age groups (Fig 3A). The onset of cardiac involvement for most patients occurred early in life (prenatal, 7/28; at birth, 16/28; during infancy, 5/28) (S1 Table). In adult respondents with cardiac issues (n = 6), onset was early in life (by infancy) for 3, in childhood for 2, and in adulthood (30s) for 1 (S1 Table). Symptoms of cardiovascular involvement included calcification in arteries/heart/aorta (n = 18), hypertension (n = 5), cardiomyopathy (n = 4), myocardial infarction (n = 3), arrhythmia (n = 3), and cardiac failure (n = 1).

### D. Skeletal/Mobility issues

Symptoms related to skeletal issues as measured by reports of bone/joint pain, impairment of mobility/fatigue, and bone/joint stiffness were predominant in the ENPP1 Deficiency cohort (Fig 3B) with 80% of the adult and pediatric participants reporting impairments in these areas. There was an increasing prevalence of skeletal/mobility issues with age (higher in children and adults than in infants). Twenty-three of 32 (72%) patients with *ENPP1* variants had bone/joint pain (Fig 3B). Of those with bone/joint pain (n = 23), the onset was early in life (0 to 5 years) for a majority: 16 (70%). In the adults (n = 7), 3 had onset of pain before 5 years of age; 3, after 20 years of age (S2 Table). Pain was frequently reported when active (9/23) and reported to be persistent at all times (7/23) (S2 Table). Nine patients had interventions to manage skeletal complications (surgery, n = 5; phosphates/calcitriol, n = 7; braces/orthotics, n = 2), while twelve had no interventions. Skeletal symptoms improved over time in 6 patients, worsened over time in 9 patients, and stayed the same in 8 patients (S2 Table).

Seventy-two percent (23/32) of patients with *ENPP1* variants reported some degree of fatigue or impaired mobility; fatigue only (n = 10), mobility impairment only (n = 3), or both (n = 10) (S3 Table). The onset of fatigue varied widely and ranged from birth to adulthood. Most reported fatigue when active (12/23 [52%]) (S3 Table). Mobility impairment onset also varied widely and ranged from birth to adulthood. Of those with fatigue and/or mobility impairment (n = 23), 14 had no interventions, while 5 had one of the following interventions (surgery, n = 3; supportive care, n = 2; physical therapy, n = 2). Not all respondents reported these data. Over time, 11 stayed the same, 6 improved, and 5 worsened (S3 Table).

### E. Burden of ENPP1 Deficiency and infant onset ABCC6 Deficiency

Based on the weighted score, fear of the unknown was the most reported burden for the infant ABCC6 Deficiency cohort, followed by physical health related to heart issues, self-care related to management of treatments, and difficulty with hospital experience (Table 4). The highest reported burden for infants with ENPP1 Deficiency based on weighted score was physical health related to heart issues, followed by self-care related to difficulty with hospital experience, and mental health related to fear of the unknown (Table 4). The highest reported burden for children with ENPP1 Deficiency was self-care related to management of treatments, followed by physical health related to hearing loss, mental health due to stress/anxiety, and physical health related to bone and joint pain (Table 4). In adults with ENPP1 Deficiency, the highest reported burden based on weighted score analysis was physical health related to bone/joint pain, followed by physical health related to mobility, fatigue, and fear of the unknown (Table 4). Patients reported that these were progressive with advancing disease.

**Table 4:** Weighted scores of patients or caregiver-reported burden of infant onset ABCC6 Deficiency and ENPP1 Deficiency^a^.

Burden^b^	Weighted Score			Total Weighted Score
	Most impactful burden	Second most impactful burden	Third most impactful burden	
**Infants/Children with ABCC6 Deficiency (n = 6)**				
Mental health related to fear of unknown^c^	3	6	1	10
Physical health related to heart issues	6	0	0	6
Self-care related to management of treatments/medications	3	2	0	5
Self-care related to difficulty with hospital experience^c^	3	2	0	5
Physical health related to breathing	3	0	0	3
Social Health—peer relationships^c^	0	2	1	3
**Infants with ENPP1 Deficiency (n = 13)**				
Physical health related to heart issues	12	6	0	18
Self-care related to difficulty with hospital experience	3	4	2	9
Mental health related to fear of unknown	6	2	1	9
Physical health related to growth and development	0	4	4	8
Physical health related to breathing	6	0	0	6
Self-Care related to management of treatments/medications	3	2	1	6
Physical health related to bone/joint pain	3	2	0	5
Physical health related to mobility	3	0	1	4
Have not experienced burdens yet^c^	0	2	1	3
Physical health related to renal (kidney impairment)	3	0	0	3
**Children with ENPP1 Deficiency (n = 12)**				
Self-care related to management of treatments/medications	3	12	0	15
Physical health related to hearing loss	6	2	1	9
Mental health related to stress/anxiety	9	0	0	9
Physical health related to bone/joint pain	6	0	1	7
Self-care related to difficulty with the hospital experience	0	4	1	5
Physical health related to mobility	0	4	1	5
Social health- peer relationships	3	0	2	5
Mental health related to fear of unknown^c^	3	0	1	4
Physical health related to treatment/test discomfort	3	0	1	4
Physical health related to renal (kidney impairment)	3	0	0	3
**Adults with ENPP1 Deficiency (n = 7)**				
Physical health related to bone/joint pain^c^	9	6	1	16
Physical health related to mobility	6	0	2	8
Physical health related to fatigue	3	2	0	5
Mental health related to fear of unknown^c^	0	2	3	5
Physical health related to treatment/test discomfort	3	0	0	3
Physical health related to heart issues	0	2	0	2

ABCC6, ATP-binding cassette subfamily C member 6; ENPP1, ectonucleotide pyrophosphatase/phosphodiesterase family member 1.

^a^The burdens reported here by were collected in an unaided manner (free form).

^b^Only the top four burdens with the highest weighted scores are provided per age group and specific deficiency.

^c^Mentioned more than once by the participant.

### F. Impact on social relationships and mental health

**Fig 4:**
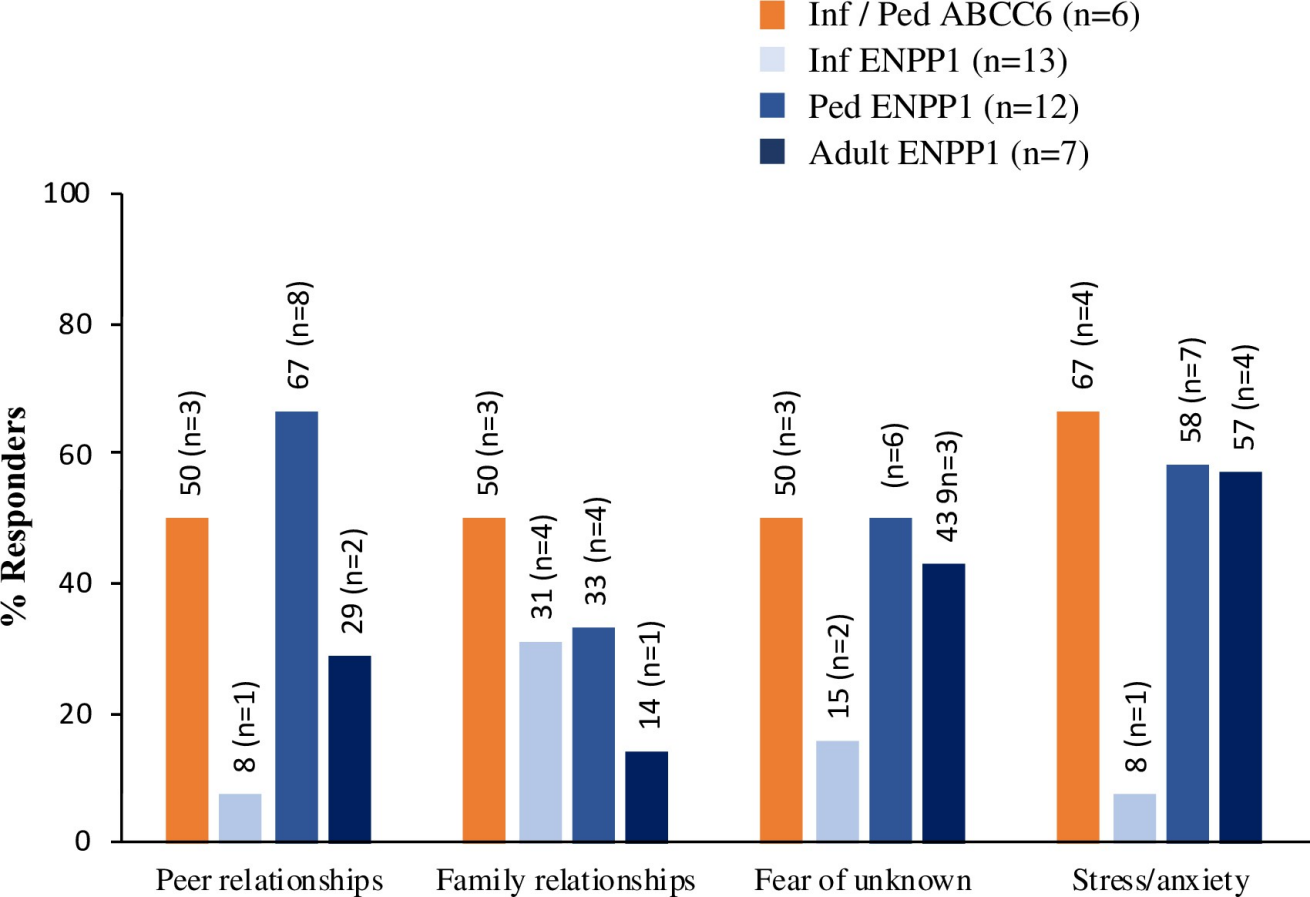
Patient/Caregiver-reported negative impact of infant onset ABCC6 Deficiency and ENPP1 Deficiency on social relationships and mental health.

Caregivers of infants with ABCC6 and ENPP1 Deficiencies reported the disorder as having an impact on social relationships and mental health of patients and/or family (Fig 4). In the infant ABCC6 Deficiency cohort, 3/6 respondents reported a negative impact on peer-to-peer and family relationships; mental health due to fear of the unknown (3/6); and stress/anxiety (4/6). Hospital visits were the most frequently mentioned reason for the impact on mental health (S5 Table). In the ENPP1 infant cohort, 4/13 caregivers of infants reported a negative impact on family relationships, due to separation of patient from family, accommodating patient’s schedule, and loss of other children due to ENPP1 Deficiency, among other reasons (Fig 4 and S4 and S5 Tables).

Caregivers of children reported the disorder as affecting peer-to-peer (8/12) and family relationships (4/12); anxiety, peer pressure due to not being able to keep up, and not feeling well were mentioned as reasons. In addition, fear of the unknown (6/12) and stress/anxiety (7/12) were reported with hospital visits/treatment mentioned as the main reason (S5 Table). In the adult cohort, 2/7 adults also reported the disorder as affecting peer-to-peer relationships due to sickness and not being able to keep up with peers (S4 Table). Fear of the unknown and stress/anxiety were reported by adults; fear of death, fertility concerns, and hospital visits were mentioned as reasons affecting mental health (Fig 4 and S4 and S5 Tables).

### G. Growth and development

Five of the six respondents for infants with ABCC6 Deficiency reported impairment of growth and development (Fig 3B). Symptoms were related to feeding (3/6), failure to thrive (2/6), short stature (2/6); some reported more than one issue (S6 Table). The use of feeding tubes (3/6) and/or high-calorie diets (1/6) was also reported (S6 Table). In infants with ENPP1 Deficiency, feeding issues (6/13) and short stature (2/13) were reported; 5/13 patients reported use of feeding tubes (Fig 3B and S6 Table).

Twenty-three of the 32 respondents with ENPP1 Deficiency reported impairment of growth and development; the age at onset of symptoms was early in life for a majority (from birth, n = 14; infancy, n = 4; childhood, n = 1) (S6 Table). The most commonly reported symptoms related to growth and development were feeding (12/32) and short stature (6/32) (S6 Table). Twelve respondents had interventions, including use of feeding tubes (n = 6), high-calorie diets (n = 4), and physical therapy (n = 1), among others. Signs and symptoms of growth and development impairment worsened in 7 patients and improved in 6; 6 stayed the same, and symptoms in 3 resolved over time (Fig 3B and S6 Table).

## Discussion

This is the first study to capture the patient experience, both subjectively in the patient voice and objectively using standardized measures of health-related quality of life to characterize the burden faced by both patients and caregivers across the age spectrum of patients with ENPP1 Deficiency or infant onset ABCC6 Deficiency. The represented population reported a high rate of mortality and cardiac complications in infants as well as multiple organ and skeletal complications in children and adults, consistent with reported disease-related complications from patient chart reviews [2,3]. This study was designed to understand how these common symptoms affected daily activities for caregivers and patients. The top reported burdens due to heart issues and fear of the unknown, are somewhat expected for caregivers of both infant cohorts because of the high mortality rate and intensive treatments with cardiac complications. As patients with ENPP1 Deficiency grew older there was a shift in the symptoms, as these patients also face hearing loss, bone/joint pain and stiffness, stress, and anxiety. However, despite overcoming the risk of mortality in early infancy, the top weighted burden reported by the caregiver remained treatment and medications, even at the age where hearing complications and skeletal complications are prevalent. Conventional treatments with phosphorus and calcitriol supplements to address the skeletal complications are burdensome and carry the potential for side effects. Oral phosphate dosages alone can be required up to 6 times/day due to its short half-life, thus creating a compliance challenge. It is possible the burden of frequent daily supplementation is a underappreciated burden to the parent and caregivers. Bone and joint pain was recognized in both the children and adult cohorts as impacting physical function with unsuccessful pain management in adults. The results suggest that while many of the morbidities are identified early in life, they can have long-term impact on patients’ performance and quality of life and pose a burden on family and caregivers. This study provides important insights into the patient/caregiver perspective on vascular/organ-related symptoms, skeletal/mobility issues, psychosocial impact, and HRQoL across the age spectrum with the use of age-specific instruments.

The PedsQL illustrates the cumulative impact of the leading burdens, as these children showed moderate to large impairment in the areas of school functioning, emotional functioning, and physical health relative to their age-matched peers. It is easy to anticipate the burden of hospitalization and management of medications as well as anxiety and stress contribute to poor emotional function in the younger children with ENPP1 Deficiency. Almost 85% of subjects reported fatigue and mobility impairment (S3 Table) as well as mild bone/joint pain which likely has an impact on physical function. In addition, struggling to keep up with schoolwork or missing school due to hospitalization or not feeling well can be an outcome of the high treatment and physical burden of the disease. The continued use of supportive medications, progressive immobility, and increase in pain contribute to overall decreased quality of life for these children.

Bone joint pain and stiffness was very prevalent in our population, something that may not be well recognized in the literature. Our results suggest there is a joint pain progression from pediatric into adult ENPP1 Deficient patients. The patient-reported burden suggests a progressive increase in prevalence of bone and joint pain (Fig 3B). Almost all the pediatric subjects reported bone/joint pain and 7/11 reported the pain location at the knees or lower limb. Pain seemed to be managed by use of acetaminophen, ibuprofen, or acetaminophen as the BPI did not record high pain severity or pain interference. In contrast, 6/7 adults reported mild to servere pain intensity with pain affecting their daily activities despite the use of pain treatments reported on the BPI scale. Five of the patients utilized pain medications without full resolution, or worsening, of pain symptoms. The pain reported in adults is similar to what has been reported in adults with x-linked hypophosphatemia [20]. Of the adults reporting pain, 3/6 described the onset of pain before the age of 5 years and 3 between the ages of 20 and 30. The difference in pain onset may be suggestive of disease progression. There is an overlap of the pain location at the knees and lower limb between the two age groups, but the adults reported additional locations, including back, shoulders, and neck as prominent locations, supporting a progressive increase in bone/joint pain. It is possible the difference in pain locations between the two age groups are due to two different mechanisms rather than a progressive pathophysiology. Pediatric patients tend to show joint calcification early in life [3] and perhaps can be managed with basic pain treatments. A recent publication characterized late-onset musculoskeletal complications, including the development of enthesis calcification, osteoarthritis, and interosseous membrane ossification [9]. The late-onset musculoskeletal complications seen in adults could have a great impact on pain interference and require more serious interventions. It is also possible that parents/caregiver proxy reporters have a different perspective of pain vs the adult who is self-reporting [21]. However, the data suggest that bone/joint pain is a progressive outcome of ENPP1 Deficiency that can have a significant impact on patients’ lives.

Patient reports of worsening symptoms suggest that ENPP1 Deficiency is a chronic and progressive disease. Few longitudinal studies related to ENPP1 Deficiency have been published to ascertain progression. However, a prospective evaluation of 16 ENPP1-deficient patients who survived past infancy found progressive hearing loss and a 100% probability of the development of rickets by the age of 14 years [2]. Furthermore, a recent natural history study of patients with the same condition shows that disease-related events increase with age, suggesting a progression of disease [3]. Taken together, the data suggest that ENPP1 Deficiency may be considered a lifelong, progressive, and unremitting disease, from the patient perspective.

This is the largest burden of illness study to date in this patient population and the study utilized well-established assessment tools validated for self-assessment. Despite ENPP1 Deficiency being a rare disease, we were able to enroll respondents to represent all age groups. The study population is small; therefore, it is difficult to determine how well it represents the overall disease population. However, we do see similar prevalence of key characteristics associated with the disease between our study population and what has been recently reported in a large history study of 84 patients with ENPP1 Deficiency [3]. Our study reported 34% mortality, 86% with cardiac issues, and 44% with rickets compared to 41%, 77%, and 49%, respectively from the natural history [3]. It seems reasonable that our study population is representative of the overall disease population. However, we reiterate that the intent of the study is not to determine a prevalence of symptoms but how symptoms can lead to burden of disease.

There are, however, other possible limitations to our study. Patients were recruited through social media and GACI Global from countries with advanced health care systems and, thus, may be more likely to participate in the study as compared to the broader population with these disorders. Additionally, the respondent-reported manifestations and their onset may not reflect objective clinical findings, as these were not verified with medical records and thus should be understood to reflect the patient/caregiver experience rather than having captured all the clinical manifestations. The survey was developed based on the understanding of the disorders from current medical literature and may not have captured all the clinical manifestations patients experience. Despite these limitations, this study provides insights into the lifelong burdens of the disorders from the patient or caregiver perspective and will help to inform treatment expectations.

## Conclusions

This study reporting on the burden of ENPP1 Deficiency and infant onset ABCC6 Deficiency from the caregiver/patient perspective expands our understanding of these disorders. ENPP1 Deficiency and infant onset ABCC6 Deficiency are debilitating diseases with lifelong morbidity, including pain and impaired mobility. Those who are affected experience impairment of quality of life and psychosocial issues throughout life. Despite best supportive care, patients across the age spectrum continue to experience significant burdens from these disorders related to the multisystem manifestations and complex medical care, and additional research is needed to further elucidate the impact on patients and caregivers.

## Supporting information

S1 Table Cardiac involvement in respondents with ABCC6/ENPP1 Deficiencies. (TIF)

S2 Table Bone/Joint pain in respondents with ABCC6 and ENPP1 Deficiencies. (TIF)

S3 Table Fatigue and mobility impairment in respondents with ABCC6 and ENPP1 Deficiencies. (TIF)

S4 Table Impact on peer-to-peer and family relationships of respondents with ABCC6 and ENPP1 Deficiencies. (TIF)

S5 Table Impact on mental health of respondents with ABCC6 and ENPP1 Deficiencies. (TIF)

S6 Table Impact on growth and development of respondents with ABCC6 and ENPP1 Deficiencies. (TIF)

S1 File (PDF)

S2 File (XLSX)

S3 File (XLSX)
