# Supplementary figures and images for: Lifelong impact of ENPP1 Deficiency and the early onset form of ABCC6 Deficiency from patient or caregiver perspective
Source: PLoS One. 2022 Jul 27;17(7):e0270632. doi: 10.1371/journal.pone.0270632 (PMC9328542; doi:10.1371/journal.pone.0270632)

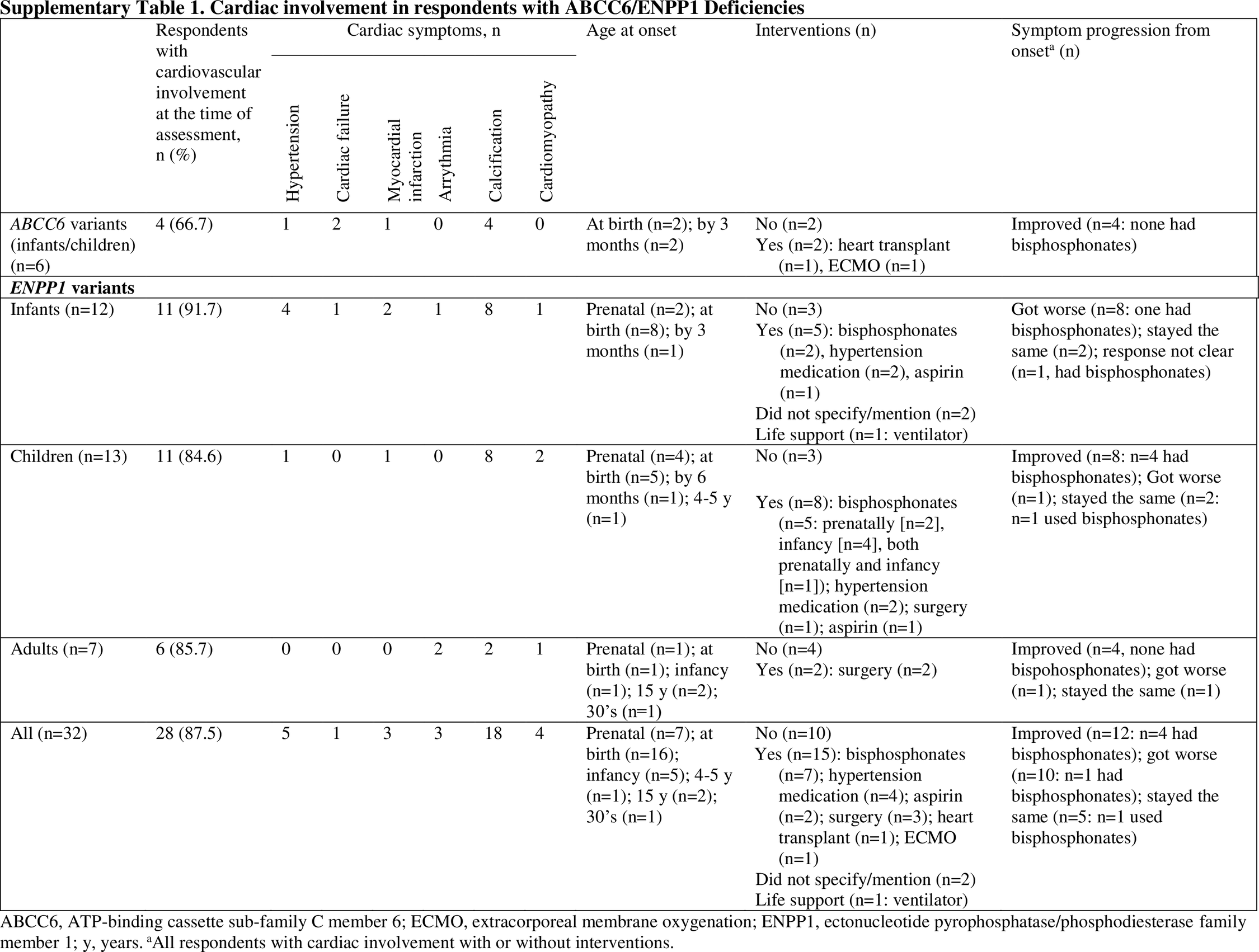

Supplement: S1 Table — (TIF) [file pone.0270632.s001.tif]

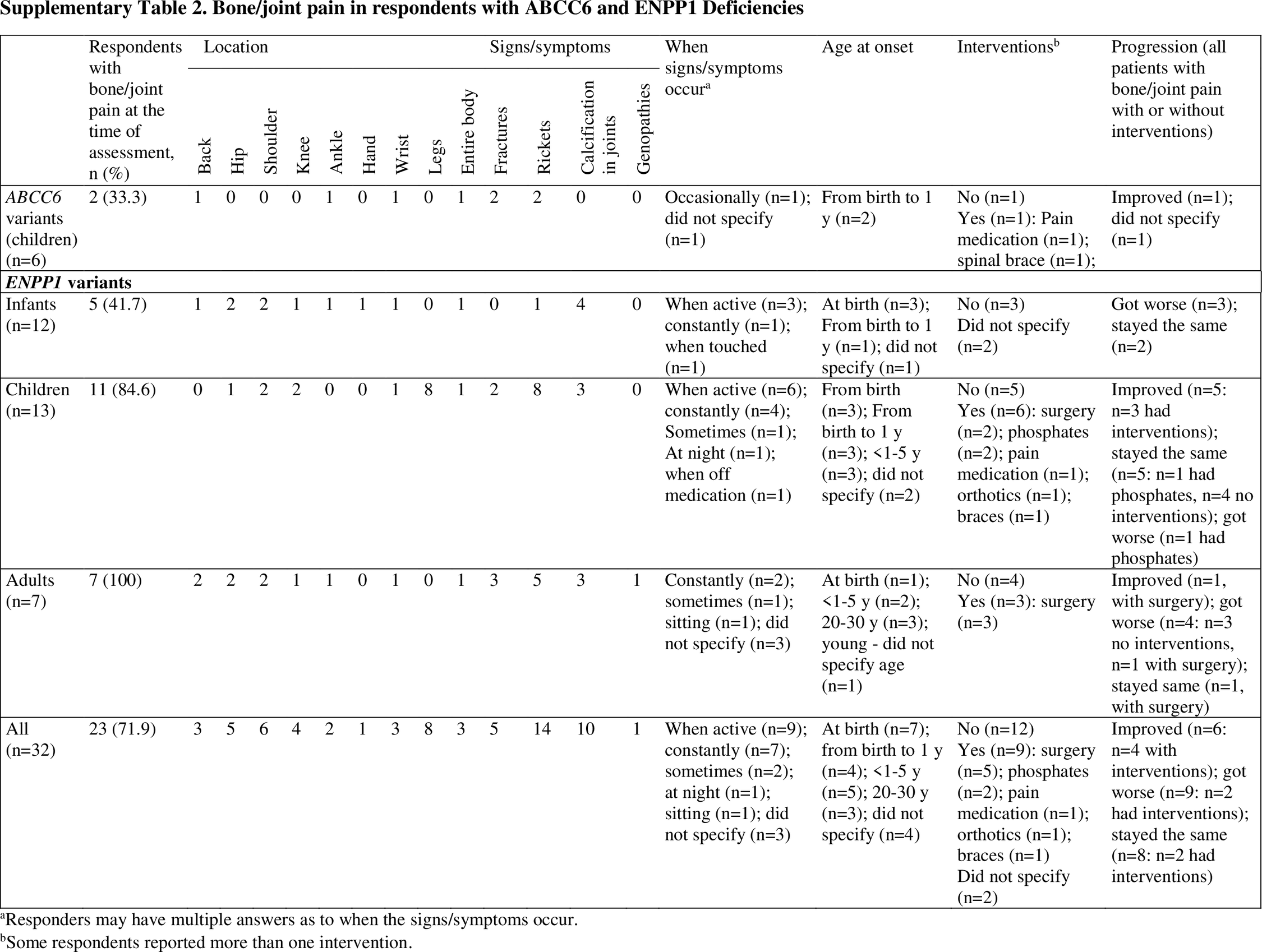

Supplement: S2 Table — (TIF) [file pone.0270632.s002.tif]

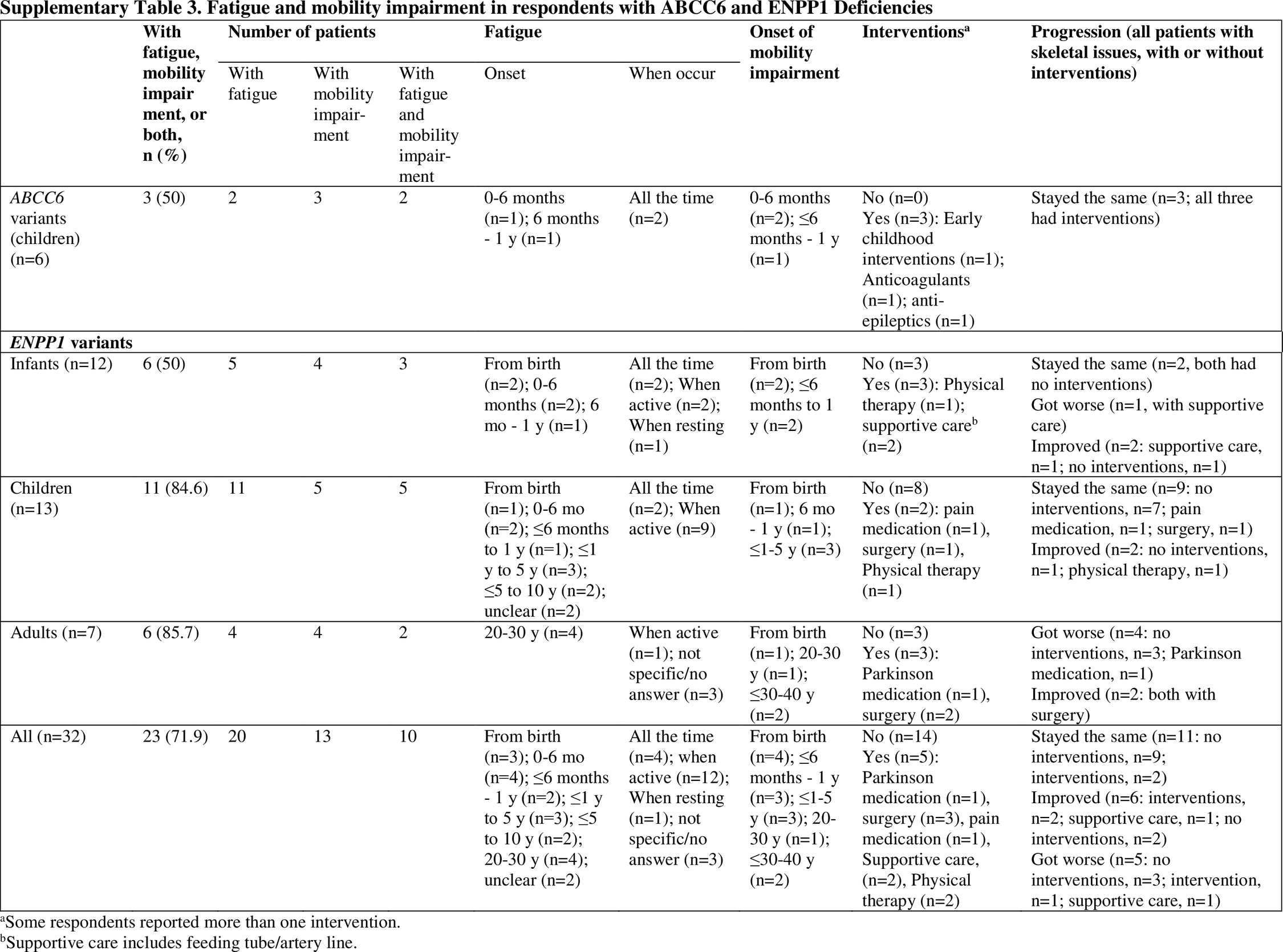

Supplement: S3 Table — (TIF) [file pone.0270632.s003.tif]

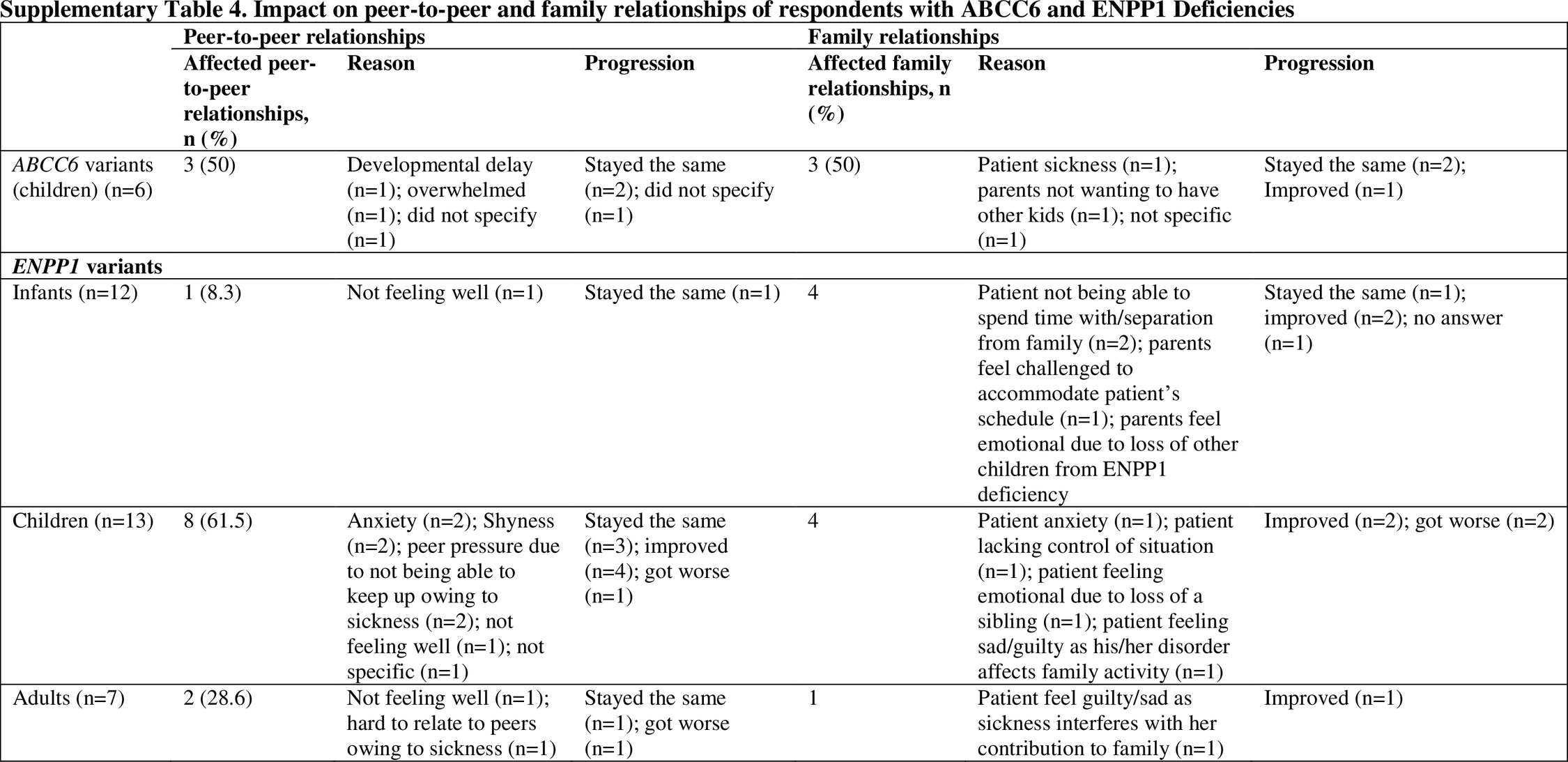

Supplement: S4 Table — (TIF) [file pone.0270632.s004.tif]

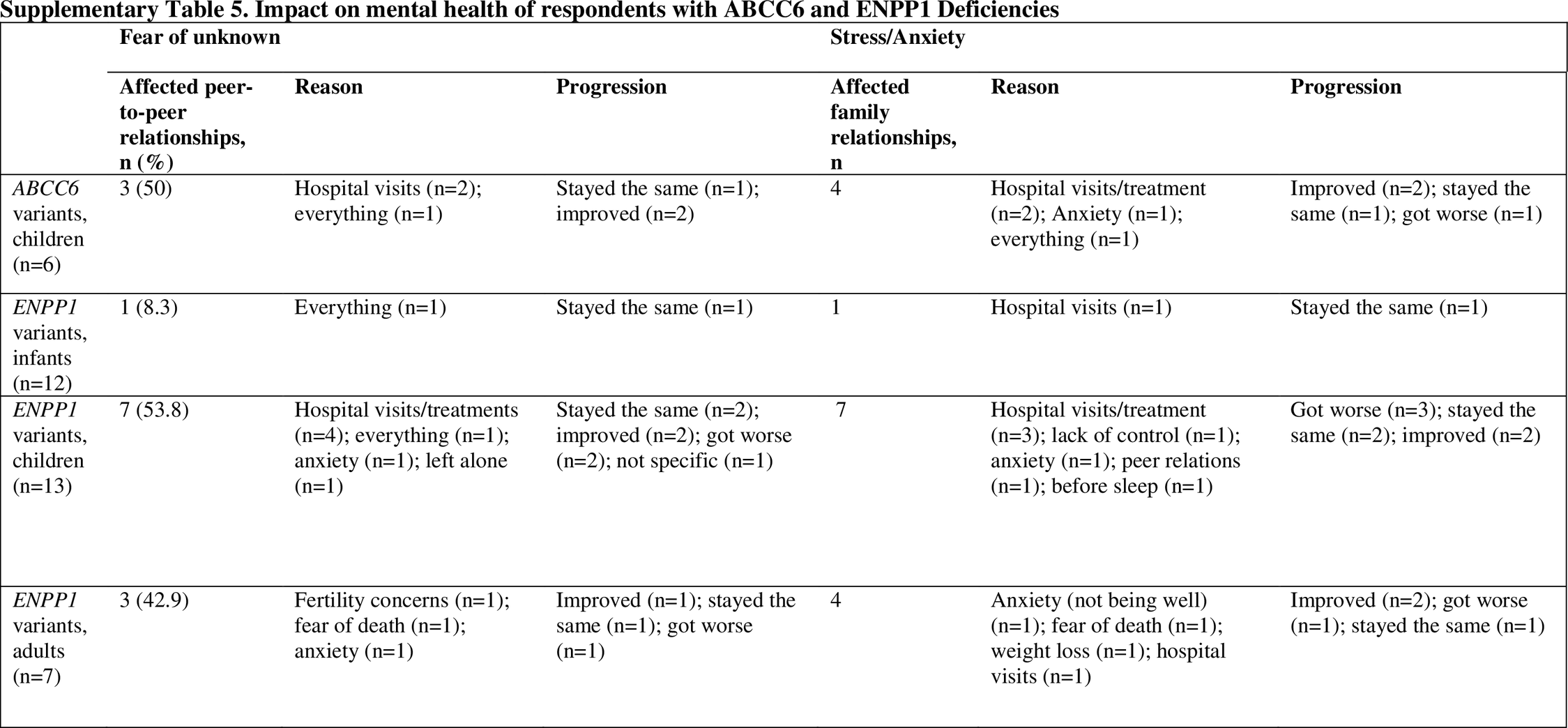

Supplement: S5 Table — (TIF) [file pone.0270632.s005.tif]

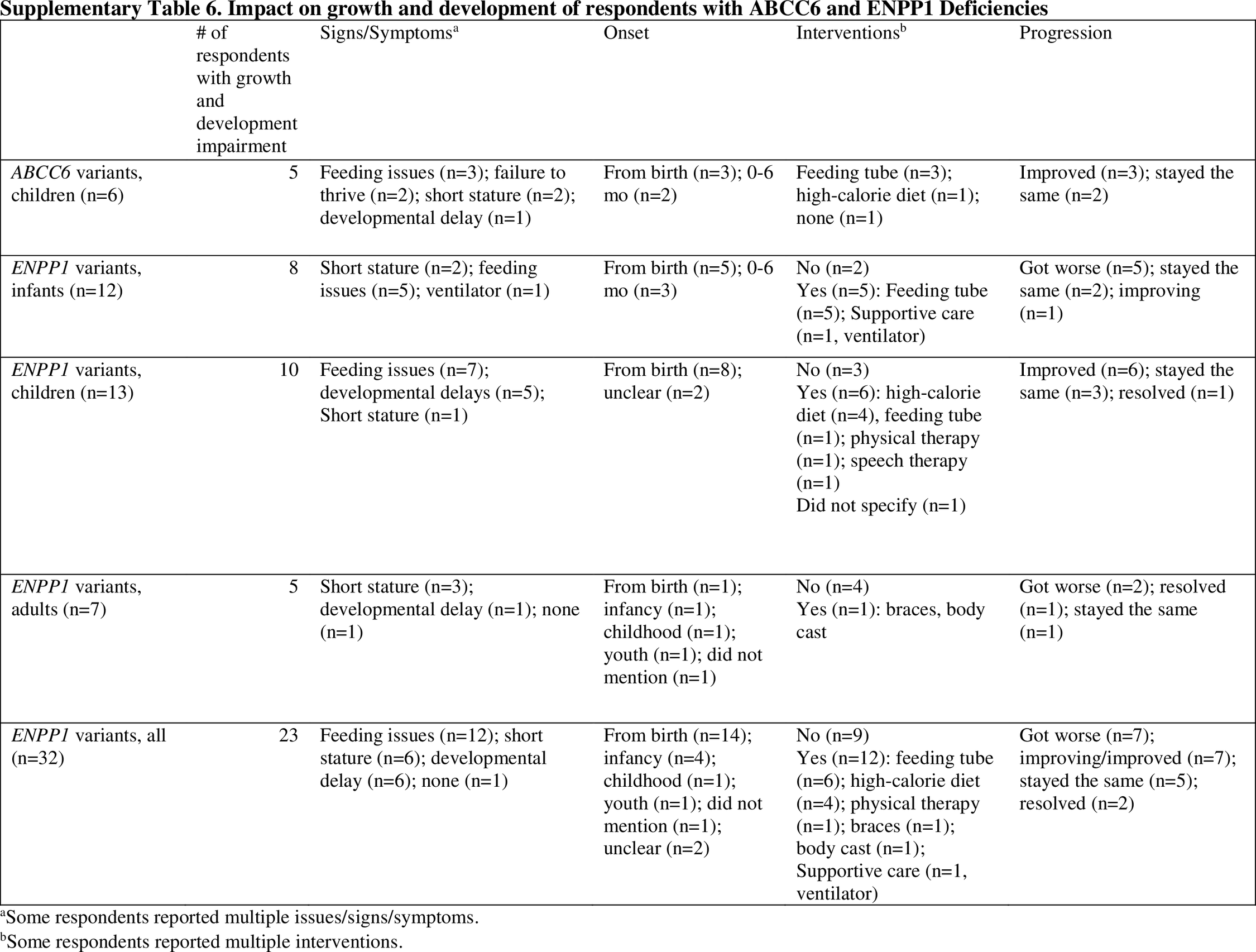

Supplement: S6 Table — (TIF) [file pone.0270632.s006.tif]
