# Supplementary material for: Lifelong impact of ENPP1 Deficiency and the early onset form of ABCC6 Deficiency from patient or caregiver perspective
Source: PLoS One. 2022 Jul 27;17(7):e0270632. doi: 10.1371/journal.pone.0270632 (PMC9328542; doi:10.1371/journal.pone.0270632)
Supplement: S1 File — (PDF) [file pone.0270632.s007.pdf]

IRB Approved at the
Study Level
Apr 22, 2020

RSVP- Understanding the Spectrum of ENPP1 deficiency and ABCC6 deficiency (GACI and ARHR2) Through the Eyes of Patients and Parents

Welcome

Thank you for your interest in the interviews regarding the burden of disease in GACI and ARHR2. GACI Global and Inozyme Pharma are conducting this study to gain a better understanding of how GACI and ARHR2 impact patients and families.

You are eligible to participate if you meet the following criteria;
* You are a patient 18 years or older, or the parent/caregiver of a patient who has been diagnosed with any form of ENPP1/ ABBC6 deficiency (GACI or ARHR2). *Please note, if you are the parent of a diagnosed child who has passed away we welcome your participation and insights*
* You are able to read, understand and sign a consent to participate in this research
* You are willing to participate in a 40 minute telephone interview, conducted in English, French or German that will take place between now and July 31, 2020.

This RSVP will take approximately 20 minutes to complete. It will allow you to provide a few days and times that will work for you to participate in a 40 minute interview. Upon completion of the RSVP questions and interview, you will be paid a $100 honorarium, in US dollars by check or gift card. There is no preparation required to participate, but you will need to be at a computer with internet connection for the interview.

To participate, proceed to the end and click "done" to submit your responses. All questions marked with an asterisk (*) are required.

Upon receipt of your RSVP someone from our staff will contact you to verify your interview.

Thank you!

Christine O'Brien
Co-President
GACI Global

Pedro Huertas, MD, PhD
Chief Medical Officer
Inozyme Pharma

RSVP- Understanding the Spectrum of ENPP1 deficiency and ABCC6 deficiency (GACI and ARHR2) Through the Eyes of Patients and Parents

Who You Are

1. Please provide the following information regarding **the person who will participate in this interview**.*

◯ Patient diagnosed with GACI or ARHR2 who is 18 years or older. Interview will cover MY experiences

◯ Parent / Caregiver of a living patient diagnosed with GACI or ARHR2 who is 18 years or older. Interview will cover MY CHILD'S experiences

◯ Parent / Caregiver of a living patient diagnosed with GACI or ARHR2 who is younger than 18 years. Interview will cover MY CHILD'S experiences

◯ Parent / Caregiver of a patient who was diagnosed with GACI or ARHR2 who has passed away. The interview will cover the experience of MY CHILD. Their year of passing is noted here:

RSVP- Understanding the Spectrum of ENPP1 deficiency and ABCC6 deficiency (GACI and ARHR2) Through the Eyes of Patients and Parents

Consent - Please Read and Scroll to Bottom for Signature

2. THIS WILL BE THE IRB APPROVED CONSENT-DRAFT CONSENT IS SHOWN BELOW

Why is this study being done?

You are being asked to be in a research study or you are being asked as the legally authorized representative, parent, or guardian to permit the subject to take part in the research. This study will try to improve understanding of the experiences of patients diagnosed with ENPP1/ABCC6 deficiency (GACI / ARHR2) and capture the burden of disease and impact of ENPP1/ABCC6 deficiency (GACI or ARHR2) on your life. Detailed data will be collected from the perspective of patients with GACI / ARHR2 or their parent/caregiver regarding their experience. This information will be used to help identify factors important to those with GACI / ARHR2.

What are the study procedures? What are my responsibilities?

If you agree to take part in this study, you will be responsible for filling out a short online RSVP questionnaire and participating in a telephone interview that will be conducted by a staff member of Engage Health, Inc., a health research vendor. As part of the RSVP, you will answer a few questions to help us understand who you are, your ethnicity, and if you qualify for the research. You will also be responsible for providing a document that ties your name (or your child's name) to a the diagnosis of any form of ENPP1/ABCC6 deficiency (GACI or ARHR2). Examples include a report of genetic testing for ENPP1/ABCC6 deficiency (GACI /ARHR2), a doctor's note, a school report or other documentation that you have on hand. We estimate that your time commitment to the RSVP will be approximately 20 minutes. You will need access to a computer and internet browser prior to the start of the interview.

If you qualify for the research, the telephone interview will be scheduled at a time that works best for you and can be conducted in English, French, or German. The interviewer will ask questions about the experiences surrounding burdens of the disease and how it has impacted your/your child's life. As you participate in the interview please know that there are no right or wrong answers. The interview is anonymous, and you will only be identified by a unique study number. If you do not qualify to participate in the research you will be notified.

We estimate that your time commitment for the RSVP questionnaire and the telephone interview will take about 60 minutes. The study will initially recruit 60 patients with 20 from each of three patient groups but there will be a minimum of 30 and a maximum of 90 participants.

What are the risks or inconveniences of the study?

Participating in this research study will not result in any clinical benefit to you. You will contribute to the understanding of this disease from your own experience and will help efforts to develop a treatment. This research study does not involve any risks. The time it takes to complete the RSVP survey and telephone interview may be a possible inconvenience. We estimate that your overall time commitment will be approximately 60 minutes (20 minutes for the RSVP/survey and 40 minutes for the telephone interview). You will be told about any new information that might change your decision to be in this study.

Engage Health, the health research vendor conducting the study, will make every reasonable effort to protect the Study Data. Submitting Personal Data over the internet always involves some risk, however. We cannot guarantee that the RSVP site and servers are 100% safe from illegal tampering or "hacking". Once Engage receives Study Data and enters it into the database, said data has the same protection that Engage Health extends to its own confidential information.

Are there costs to participate?

There are no costs to participate in this study.

What are the benefits of the study?

You may not receive a direct benefit if you agree to participate. However, we hope your participation in this study may provide a better understanding of how ENPP1/ABCC6 deficiency (GACI or ARHR2) impacts your child's life/your life in meaningful ways to you and possibly help in developing a treatment for this condition. People in the future may benefit from the information obtained from this research.

Your alternative is to not participate in this study.

How will my personal information be protected?

The interview data collected by the research vendor, Engage Health, will be stored in a locked / secure location and stored on secure, encrypted, and wholly-owned servers. Your records will be "pseudonymized" which means that your name and any information that could identify you will be removed and replaced with a unique code. The answers that you provide will be combined with those of others participating in the study and summarized in a final report. The final report will be shared with others but will not identify you because the survey is anonymous during analysis of the data. At the end of this study, the researchers may publish the results of this research. We will keep your name and other identifying information confidential, however.

To review the privacy and data storage policies of Engage Health, Inc. for the U.S. and areas outside of the U.S. please visit https://www.engagehealth.com/privacy-policy/.

Who can answer my questions about this research?

Take as much time as you like before you decide to participate in this study. We will be happy to answer any questions you have about this study.

Contact Pedro Huertas, MD, PhD, Chief Medical Officer at Inozyme, at (978) 394-5700 for questions, concerns or complaints about the research or if you think you have been harmed as a result of joining this research.

Contact the Western Institutional Review Board (WIRB) if you have questions about your rights as a research subject, concerns, complaints or input: 1-800-562-4789. WIRB is a group of people who perform an independent review of research.

What happens to the information collected for this research?

The study staff at Engage Health, Inc. may share the records generated from this research with other staff at Engage Health on an as-needed basis, and the IRB. This information is shared so the research can be conducted and properly monitored. The people receiving this information are required to protect it and your information may not be redisclosed without your permission. If you do not provide permission to use your information you cannot be in the study. As noted above, the answers that you provide will be combined with those of others participating in the study and summarized in a final report. The final report will be shared with others but will not identify you because the survey is anonymous for the data analysis. At the end of this study, the researchers may publish the results of this research. However, we will keep your name and other identifying information confidential.

This permission will not end unless you cancel it. You may cancel it by sending written notice to Engage Health, the research vendor at pengel@engagehealth.com or Attn: P. Engel, Engage Health Inc., 3265 Lexington Ave. So, Eagan MN 55121. Alternatively, you can contact Engage Health's Data Protection Officer at DataProtectionOfficer@engagehealth.com to remove your information. Any information collected before you withdraw may still be used.

A description of this clinical trial will be available on http://www.ClinicalTrials.gov, as required by U.S. Law. This Web site will not include information that can identify you. At most, the Web site will include a summary of the results. You can search this Web site at any time.

Can I stop being in the study?

Your decision to be in this study is voluntary. You will not be penalized or lose benefits if you decide not to participate or if you decide to stop participating.

Can I be removed from this research without my approval?

Your part in this study may be stopped at any time by Engage Health, Inc., the health research vendor, or the sponsor without your approval for any reason, including:

- if it is in your best interest;
- you do not consent to potential changes made in the study plan (if applicable); or
- you do not keep your scheduled appointment for the telephone interview.

Will I be paid for taking part in this research?

If you qualify and participate in the RSVP survey and the interview, you will be paid a $100 honorarium as compensation for your time. You will have two choices regarding how you will be paid. You can either be paid in US dollars by a check that will be mailed to you by Engage Health at the completion of your interview, or you can choose to be paid by an electronic "e-gift card" to Amazon.com, that will be emailed to you by Engage Health at the completion of your interview.

If the telephone interview is not completed for any reason, you will not receive the honorarium.

Documentation of Consent

I have read this form and decided that I will participate in the research study described above. Its general purposes, particulars of involvement and possible risks and inconveniences have been explained to my satisfaction. I understand that my participation is voluntary, and I can withdraw at any time.

*

	Initials	Date
Please Provide:		

RSVP- Understanding the Spectrum of ENPP1 deficiency and ABCC6 deficiency (GACI and ARHR2) Through the Eyes of Patients and Parents

Assent - Please Read and Scroll to Bottom for Signature

3. **As part of this study, you will be asked to print and fill out the "Brief Pain Inventory". If your child is between 12 and 18 years of age, and will participate in filling this out, they must read and sign the Assent Form below. If they will not participate, skip to the next page.**

THIS WILL BE THE IRB APPROVED ASSENT-DRAFT ASSENT IS SHOWN BELOW

Assent Form
(For participants age 12-18 years)

As part of this study, you will be asked to print and fill out a Brief Pain Inventory. If your child will participate in filling this out, they must read and sign the Assent Form.

Sponsor: Inozyme Pharma

Protocol Number: Inozyme0001
WIRB® Protocol #_____

Protocol Title: Understanding the Spectrum of ENPP1 deficiency and ABCC6 deficiency (GACI and ARHR2) Through the Eyes of Patients and Parents; Burden of Illness Perspectives from Patients and Parents who Speak English, French or German

Protocol Version and Date: Version 1.0, April 6, 2020

Principal Investigator:
Pedro Huertas, MD, PhD
Chief Medical Officer, Inozyme Pharma

Christine O'Brien
Co-President of GACI Global

Daytime Telephone Number:

Dr. Huertas (978)-394-5700

GACI Global (777)-754-0912

You are being invited to take part in a research study. A person who takes part in a research study is called a research subject, or research participant.

This research study is about things people with ENPP1 deficiency or ABCC6 deficiency (also called GACI or ARHR2) or their parent (caregiver) may be most interested in, based on the severity of the disease. The study will enroll between 30 and 90 participants.

There are some things about this research study you should know. You and your parent will fill out a short online questionnaire that will take about 5 minutes. You or your parent (caregiver) will print off

two short questionnaires that you will fill out, which will take about 15 minutes. You will upload the completed questionnaires and a document that proves that you have ENPP1 deficiency or ABCC6 deficiency. We will then set up a telephone interview with your parent. The telephone interview will take about 40 minutes. As you fill out the questionnaire , please know there are no right or wrong answers. Your answers are anonymous which means the results will not tell people who you are.

There are no known physical risks associated with being in this research study. A risk is when something might be unsafe for you. It will take about one hour to complete all of these activities, but about 15 minutes to participate in your part, the filling of the questionnaires. The health research company that is collecting the study information will make every reasonable effort to protect the information you provide. However, there is always some risk that the computer system could be tampered with or "hacked".

You may not benefit and there is no intended benefit to your health if you agree to participate. Benefit means something good happens to you. Benefits might be that researchers who are looking for new treatments for ENPP1 deficiency or ABCC6 deficiency will know more about how people with these conditions and their parents (caregivers) want those treatments to work. People in the future may benefit from the study results.

Please talk this over with your parents before you decide whether or not to participate. We will also ask your parents to give their permission for you to take part in this study. But even if your parents say "yes" you can still decide not to do this.

You do not have to be in this study if you do not want to be. If you decide to stop after we begin, that's okay too. If this happens you can ask us to destroy any answers you already gave us.

When we are finished with this study your answers will be added to the answers of other people in the study and we will write a report about what was learned. This report will not include your name or that you were in the study. The people who are doing this study may publish it in a medical journal to teach others more about the things that people with ENPP1 deficiency or ABCC6 deficiency and their parents (caregivers) care about.

You can ask any questions that you have about the study. If you have a question later that you didn't think of now, you can call Dr. Pedro Huertas, Principal Investigator, at (978)-394-5700 or reach him by email at pedro.huertas@inozyme.com. You could also reach Christine O'Brien, the Co-President of GACI Global, and the Co-Investigator for the study, at (777)-754-0912 or email christineobrien@gaciglobal.org.

Your parents know about the study too.

Assent

If you would like to take part, please initial and date this form below. You and your parents (caregiver) can print out a copy of this form if you want to keep it. To do this, right click anywhere on the page and select print from the drop down menu. This will pop up your printer menu and you can print a copy.

	Initials	Date
Please Provide:		

RSVP- Understanding the Spectrum of ENPP1 deficiency and ABCC6 deficiency (GACI and ARHR2) Through the Eyes of Patients and Parents

About You

4. Please provide the following information in order that we can schedule an interview time for you, contact you for the interview, and process your honorarium at the completion of the interview.
   *
   Please note that by providing your contact information you are providing authorization to Engage Health, Inc. that they may contact you regarding this research study

First Name:	
Last Name:	
Email Address:	
Street Address (where to send your honoraria check/gift card):	
City:	
State or Province: (e.g. NY):	
Zip or Postal Code:	
Country:	
Telephone Number at Which to Contact You for the Interview: (with area or country code)	

5. How did you learn about this research?
   - ◯ Facebook or Other Social Media Post
   - ◯ Communication from Patient Association
   - ◯ Communication from Engage Health
   - ◯ Through a family member or friend
   - ◯ From my healthcare provider
   - ◯ From GACI Global
   - ◯ Other, please specify

The following questions pertain to the person in your family who has been diagnosed with GACI or ARHR2 who will be the subject of this interview.

6. Please share with us the following information about you/your child (the person in your family who was diagnosed with GACI or ARHR2 and who will be the subject of this interview):

   Note, if your child has passed away, please answer the questions related to your child before their passing*

Year of Birth:	
Gender at Birth:	
Current Gender:	
Diagnosis (GACI or ARHR2):	

Year of diagnosis: [ ]

7. Please select your / your child's age range (the subject of this interview). *

- ◯ Birth to 24 months - parent interview
- ◯ 25-36 months, - parent interview
- ◯ 3+ to 4 years - parent interview
- ◯ 5 to 7 years - parent interview
- ◯ 8 to 12 years - parent interview
- ◯ 13 to 18 years - parent interview
- ◯ 18 to 25 - parent interview
- ◯ 26 and older - parent interview

8. In this question you will be asked to share information about the age of your child. This information is used to provide a Quality of Life Questionnaire that you will fill out, which is based on DEVELOPMENTAL AGE.

IF YOUR CHILD IS 4 YEARS OR YOUNGER, AND <u>WAS NOT BORN PREMATURELY</u>, SIMPLY SELECT THE OPTION THAT BEST DESCRIBES THE AGE OF YOUR CHILD.

IF YOUR CHILD IS 4 YEARS OR YOUNGER, AND <u>WAS BORN MORE THAN 3 WEEKS BEFORE THE FULL TERM DATE OF DELIVERY (DUE DATE)</u>, YOU WILL CALCULATE AS FOLLOWS;

1. CONSIDER YOUR CHILD'S CURRENT AGE
2. SUBTRACT THE NUMBER OF WEEKS OF PREMATURITY FROM YOUR CHILD'S AGE

(For example, if your child is currently 36 months old, but was born 5 weeks prematurely they would be considered 34 months 3 weeks old)

3. THEN SELECT THE OPTION THAT BEST DESCRIBES THE AGE OF YOUR CHILD

- ◯ Birth up to but not including one month
- ◯ 1 month 0 days through 2 months 30 days
- ◯ 3 months 0 days through 4 months 30 days
- ◯ 5 months 0 days through 6 months 30 days
- ◯ 7 months 0 days through 8 months 30 days
- ◯ 9 months 0 days through 10 months 30 days
- ◯ 11 month 0 days through 12 months 30 days
- ◯ 13 month 0 days through 14 months 30 days
- ◯ 15 months 0 days through 16 months 30 days
- ◯ 17 months 0 days through 18 months 30 days
- ◯ 19 months 0 days through 20 months 30 days
- ◯ 21 months 0 days through 22 months 30 days
- ◯ 23 months 0 days through 25 months 15 days
- ◯ 25 months 16 days through 28 months 15 days
- ◯ 28 months 16 days through 31 months 15 days
- ◯ 31 months 16 days through 34 months 15 days
- ◯ 34 months 16 days through 35 months 30 days
- ◯ 36 months through 48 months

Ages and Stages

In this section you will be given a link to a document. Click the link for it to download onto your computer. Please print the document and fill it out, writing as legibly as possible. When you are done, hit next to move to the next page.

Please click the link below to download and then print the Ages and Stages Questionnaire for your child who is 1 month 0 days through 2 months 30 days old.

Ages and Stages 2 Month Questionnaire

If the above hyperlink does not work, you can copy and paste the following into your internet browser: https://www.engagehealth.com/survey/UploadedImageDisplayFS.aspx?path=C:\inetpub\wwwroot\survey\UploadedImages\ASQ3_2mo_Eng.pdf

Please click the link below to download and then print the Ages and Stages Questionnaire for your child who is 3 months 0 days through 4 months 30 days old.

Ages and Stages 4 Month Questionnaire

If the above hyperlink does not work, you can copy and paste the following into your internet browser: https://www.engagehealth.com/survey/UploadedImageDisplayFS.aspx?path=C:\inetpub\wwwroot\survey\UploadedImages\ASQ3_4mo_Eng.pdf

Please click the link below to download and then print the Ages and Stages Questionnaire for your child who is 5 months 0 days through 6 months 30 days old.

Ages and Stages 6 Month Questionnaire

If the above hyperlink does not work, you can copy and paste the following into your internet browser: https://www.engagehealth.com/survey/UploadedImageDisplayFS.aspx?path=C:\inetpub\wwwroot\survey\UploadedImages\ASQ3_6mo_Eng.pdf

Please click the link below to download and then print the Ages and Stages Questionnaire for your child who is 7 months 0 days through 8 months 30 days old.

Ages and Stages 8 Month Questionnaire

If the above hyperlink does not work, you can copy and paste the following into your internet browser: https://www.engagehealth.com/survey/UploadedImageDisplayFS.aspx?path=C:\inetpub\wwwroot\survey\UploadedImages\ASQ3_8mo_Eng.pdf

Please click the link below to download and then print the Ages and Stages Questionnaire for your child who is 9 months 0 days through 10 months 30 days old.

Ages and Stages 10 Month Questionnaire

If the above hyperlink does not work, you can copy and paste the following into your internet browser: https://www.engagehealth.com/survey/UploadedImageDisplayFS.aspx?path=C:\inetpub\wwwroot\survey\UploadedImages\ASQ3_10mo_Eng.pdf

Please click the link below to download and then print the Ages and Stages Questionnaire for your child who is 11 month 0 days through 12 months 30 days old.

Ages and Stages 12 Month Questionnaire

If the above hyperlink does not work, you can copy and paste the following into your internet browser: https://www.engagehealth.com/survey/UploadedImageDisplayFS.aspx?path=C:\inetpub\wwwroot\survey\UploadedImages\ASQ3_12mo_Eng.pdf

Please click the link below to download and then print the Ages and Stages Questionnaire for your child who is 13 month 0 days through 14 months 30 days old.

Ages and Stages 14 Month Questionnaire

If the above hyperlink does not work, you can copy and paste the following into your internet browser: https://www.engagehealth.com/survey/UploadedImageDisplayFS.aspx?path=C:\inetpub\wwwroot\survey\UploadedImages\ASQ3_14mo_Eng.pdf

Please click the link below to download and then print the Ages and Stages Questionnaire for your child who is 15 months 0 days through 16 months 30 days old.

Ages and Stages 16 Month Questionnaire

If the above hyperlink does not work, you can copy and paste the following into your internet browser: https://www.engagehealth.com/survey/UploadedImageDisplayFS.aspx?path=C:\inetpub\wwwroot\survey\UploadedImages\ASQ3_16mo_Eng.pdf

Please click the link below to download and then print the Ages and Stages Questionnaire for your child who is 17 months 0 days through 18 months 30 days old.

Ages and Stages 18 Month Questionnaire

If the above hyperlink does not work, you can copy and paste the following into your internet browser: https://www.engagehealth.com/survey/UploadedImageDisplayFS.aspx?path=C:\inetpub\wwwroot\survey\UploadedImages\ASQ3_18mo_Eng.pdf

Please click the link below to download and then print the Ages and Stages Questionnaire for your child who is 19 months 0 days through 20 months 30 days old.

Ages and Stages 20 Month Questionnaire

If the above hyperlink does not work, you can copy and paste the following into your internet browser: https://www.engagehealth.com/survey/UploadedImageDisplayFS.aspx?path=C:\inetpub\wwwroot\survey\UploadedImages\ASQ3_20mo_Eng.pdf

Please click the link below to download and then print the Ages and Stages Questionnaire for your child who is 21 months 0 days through 22 months 30 days old.

Ages and Stages 22 Month Questionnaire

If the above hyperlink does not work, you can copy and paste the following into your

internet
browser: https://www.engagehealth.com/survey/UploadedImageDisplayFS.aspx?path=C:\inetpub\wwwroot\survey\UploadedImages\ASQ3_22mo_Eng.pdf

Please click the link below to download and then print the Ages and Stages Questionnaire for your child who is 23 months 0 days through 25 months 15 days old.

Ages and Stages 24 Month Questionnaire

If the above hyperlink does not work, you can copy and paste the following into your internet
browser: https://www.engagehealth.com/survey/UploadedImageDisplayFS.aspx?path=C:\inetpub\wwwroot\survey\UploadedImages\ASQ3_24mo_Eng.pdf

Please click the link below to download and then print the Ages and Stages Questionnaire for your child who is 25 months 16 days through 28 months 15 days old.

Ages and Stages 27 Month Questionnaire

If the above hyperlink does not work, you can copy and paste the following into your internet
browser: https://www.engagehealth.com/survey/UploadedImageDisplayFS.aspx?path=C:\inetpub\wwwroot\survey\UploadedImages\ASQ3_27mo_Eng.pdf

Please click the link below to download and then print the Ages and Stages Questionnaire for your child who is 28 months 16 days through 31 months 15 days old.

Ages and Stages 30 Month Questionnaire

If the above hyperlink does not work, you can copy and paste the following into your internet
browser: https://www.engagehealth.com/survey/UploadedImageDisplayFS.aspx?path=C:\inetpub\wwwroot\survey\UploadedImages\ASQ3_30mo_Eng.pdf

Please click the link below to download and then print the Ages and Stages Questionnaire for your child who is 31 months 16 days through 34 months 15 days old.

Ages and Stages 33 Month Questionnaire

If the above hyperlink does not work, you can copy and paste the following into your internet
browser: https://www.engagehealth.com/survey/UploadedImageDisplayFS.aspx?path=C:\inetpub\wwwroot\survey\UploadedImages\ASQ3_33mo_Eng.pdf

Please click the link below to download and then print the Ages and Stages Questionnaire for your child who is 34 months 16 days through 35 months 30 days old.

Ages and Stages 36 Month Questionnaire

If the above hyperlink does not work, you can copy and paste the following into your internet
browser: https://www.engagehealth.com/survey/UploadedImageDisplayFS.aspx?path=C:\inetpub\wwwroot\survey\UploadedImages\ASQ3_36mo_Eng.pdf

RSVP- Understanding the Spectrum of ENPP1 deficiency and ABCC6 deficiency (GACI and ARHR2) Through the Eyes of Patients and Parents

PEDs QL

In this section you will be given a link to a document. Choose the document that most closely corresponds with where you live: United States/Canada, United Kingdom or Australia. Click the link for it to download onto your computer. Please print the document and fill it out, writing as legibly as possible. When you are done, hit next to move to the next page.

English version for United States/Canada
Please click the link below to download and then print the PEDs QL Questionnaire for parents of children 2 - 4 years old. If the hyperlink does not work, please copy and paste it into your browser.

https://www.engagehealth.com/survey/UploadedImageDisplayFS.aspx?path=C:\inetpub\wwwroot\survey\UploadedImages\PedsQL-4.0-Core-PT_AU4.0_eng-USori_.pdf

English version for United Kingdom
Please click the link below to download and then print the PEDs QL Questionnaire for parents of children 2 - 4 years old. If the hyperlink does not work, please copy and paste it into your browser.

https://www.engagehealth.com/survey/UploadedImageDisplayFS.aspx?path=C:\inetpub\wwwroot\survey\UploadedImages\PedsQL-4.0-Core-PT_AU4.0_eng-GB2.pdf

English version for Australia
Please click the link below to download and then print the PEDs QL Questionnaire for parents of children 2 - 4 years old. If the hyperlink does not work, please copy and paste it into your browser.

https://www.engagehealth.com/survey/UploadedImageDisplayFS.aspx?path=C:\inetpub\wwwroot\survey\UploadedImages\PedsQL-4.0-Core-PT_AU4.0_eng-AU3.pdf

English version for United States/Canada
Please click the link below to download and then print the PEDs QL Questionnaire for parents of children 5 - 7 years old. If the hyperlink does not work, please copy and paste it into your browser.

https://www.engagehealth.com/survey/UploadedImageDisplayFS.aspx?path=C:\inetpub\wwwroot\survey\UploadedImages\PedsQL-4.0-Core-PYC_AU4.0_eng-USori.pdf

English version for United Kingdom
Please click the link below to download and then print the PEDs QL Questionnaire for parents of children 5 - 7 years old. If the hyperlink does not work, please copy and paste it into your browser.

https://www.engagehealth.com/survey/UploadedImageDisplayFS.aspx?path=C:\inetpub\wwwroot\survey\UploadedImages\PedsQL-4.0-Core-PYC_AU4.0_eng-GB2.pdf

English version for Australia
Please click the link below to download and then print the PEDsQL Questionnaire for parents of children 5 - 7 years old. If the hyperlink does not work, please copy and paste it into your browser.

https://www.engagehealth.com/survey/UploadedImageDisplayFS.aspx?path=C:\inetpub\wwwroot\survey\UploadedImages\PedsQL-4.0-Core-PYC_AU4.0_eng-AU3.pdf

English version for United States/Canada
Please click the link below to download and then print the PEDs QL Questionnaire for parents of children 8 - 12 years old. If the hyperlink does not work, please copy and paste it into your browser.

https://www.engagehealth.com/survey/UploadedImageDisplayFS.aspx?path=C:\inetpub\wwwroot\survey\UploadedImages\PedsQL-4.0-Core-PC_AU4.0_eng-USori.pdf

English version for United Kingdom
Please click the link below to download and then print the PEDs QL Questionnaire for parents of children 8 - 12 years old. If the hyperlink does not work, please copy and paste it into your browser.

https://www.engagehealth.com/survey/UploadedImageDisplayFS.aspx?path=C:\inetpub\wwwroot\survey\UploadedImages\PedsQL-4.0-Core-PC_AU4.0_eng-GB2.pdf

English version for Australia
Please click the link below to download and then print the PEDs QL Questionnaire for parents of children 8 - 12 years old. If the hyperlink does not work, please copy and paste it into your browser.

https://www.engagehealth.com/survey/UploadedImageDisplayFS.aspx?path=C:\inetpub\wwwroot\survey\UploadedImages\PedsQL-4.0-Core-PC_AU4.0_eng-AU3.pdf

English version for United States/Canada
Please click the link below to download and then print the PEDs QL Questionnaire for parents of children 13 - 18 years old. If the hyperlink does not work, please copy and paste it into your browser.

https://www.engagehealth.com/survey/UploadedImageDisplayFS.aspx?path=C:\inetpub\wwwroot\survey\UploadedImages\PedsQL-4.0-Core-PA_AU4.0_eng-USori.pdf

English version for United Kingdom
Please click the link below to download and then print the PEDs QL Questionnaire for parents of children 13 - 18 years old. If the hyperlink does not work, please copy and paste it into your browser.

https://www.engagehealth.com/survey/UploadedImageDisplayFS.aspx?path=C:\inetpub\wwwroot\survey\UploadedImages\PedsQL-4.0-Core-PA_AU4.0_eng-GB2.pdf

English version for Australia
Please click the link below to download and then print the PEDs QL Questionnaire for parents of children 13 - 18 years old. If the hyperlink does not work, please copy and paste it into your browser.

https://www.engagehealth.com/survey/UploadedImageDisplayFS.aspx?path=C:\inetpub\wwwroot\survey\UploadedImages\PedsQL-4.0-Core-PA_AU4.0_eng-AU3.pdf

English version for United States/Canada
Please click the link below to download and then print the PEDs QL Questionnaire for parents of children 18 - 25 years old. If the hyperlink does not work, please copy and paste it into your browser.

https://www.engagehealth.com/survey/UploadedImageDisplayFS.aspx?path=C:\inetpub\wwwroot\survey\UploadedImages\PedsQL-4.0-Core-PYA_AU4.0_eng-USori.pdf

English version for United Kingdom
Please click the link below to download and then print the PEDs QL Questionnaire for parents of children 18 - 25 years old. If the hyperlink does not work, please copy and paste it into your browser.

https://www.engagehealth.com/survey/UploadedImageDisplayFS.aspx?path=C:\inetpub\wwwroot\survey\UploadedImages\PedsQL-4.0-Core-PYA_AU4.0_eng-GB.pdf

English version for Australia
Please click the link below to download and then print the PEDs QL Questionnaire for parents of children 18 - 25 years old. If the hyperlink does not work, please copy and paste it into your browser.

https://www.engagehealth.com/survey/UploadedImageDisplayFS.aspx?path=C:\inetpub\wwwroot\survey\UploadedImages\PedsQL_4.0-Core-PYA_AU4.0_eng-AU.pdf

English version for United States/Canada
Please click the link below to download and then print the PEDs QL Questionnaire for parents of children 25+ years old. If the hyperlink does not work, please copy and paste it into your browser.

https://www.engagehealth.com/survey/UploadedImageDisplayFS.aspx?path=C:\inetpub\wwwroot\survey\UploadedImages\PedsQL-4.0-Core-PAdult_AU4.0_eng-USori.pdf

English version for United Kingdom
Please click the link below to download and then print the PEDs QL Questionnaire for parents of children 25+ years old. If the hyperlink does not work, please copy and paste it into your browser.

https://www.engagehealth.com/survey/UploadedImageDisplayFS.aspx?path=C:\inetpub\wwwroot\survey\UploadedImages\PedsQL-4.0-Core-PAdult_AU4.0_eng-GB.pdf

English version for Australia
Please click the link below to download and then print the PEDs QL Questionnaire for parents of children 25+ years old. If the hyperlink does not work, please copy and paste it into your browser.

https://www.engagehealth.com/survey/UploadedImageDisplayFS.aspx?path=C:\inetpub\wwwroot\survey\UploadedImages\PedsQL-4.0-Core-PAdult_AU4.0_eng-AU.pdf

RSVP- Understanding the Spectrum of ENPP1 deficiency and ABCC6 deficiency (GACI and ARHR2) Through the Eyes of Patients and Parents

PROMIS

In this section you will be given a link to a document. Click the link for it to download onto your computer. Please print the document and fill it out, writing as legibly as possible. When you are done, hit next to move to the next page.

Please click the link below to download and then print the PROMIS Questionnaire.

PROMIS Questionnaire

If the above hyperlink does not work, you can copy and paste the following into your internet browser: https://www.engagehealth.com/survey/UploadedImageDisplayFS.aspx?path=C:\inetpub\wwwroot\survey\UploadedImages\PROMIS_Custom_Short_Form_v2.0__Physical_Function_12-item_INZ-ENPP1_26Mar2020.pdf

RSVP- Understanding the Spectrum of ENPP1 deficiency and ABCC6 deficiency (GACI and ARHR2) Through the Eyes of Patients and Parents

The Brief Pain Inventory

In this section you will see a link to the Brief Pain Inventory, which captures information about your/your child's experience with pain related to GACI or ARHR2. Click the link for it to download onto your computer. Please print the document and fill it out, writing as legibly as possible. When you are done, hit next to move to the next page.

Please click the link below to download and then print the Brief Pain Index Questionnaire.

Brief Pain Index Questionnaire

If the above hyperlink does not work, you can copy and paste the following into your internet browser: https://www.engagehealth.com/survey/UploadedImageDisplayFS.aspx?path=C:\inetpub\wwwroot\survey\UploadedImages\BPI-SF_English_INZ_ENPP1.pdf

RSVP- Understanding the Spectrum of ENPP1 deficiency and ABCC6 deficiency (GACI and ARHR2) Through the Eyes of Patients and Parents

About You

9. Please select the options that will help us understand you/your child's ethnicity.*

Please select all that apply.

- ☐ White / Caucasian:
- ☐ Hispanic / Latino or Spanish:
- ☐ Black / African American:
- ☐ Asian or Asian Indian:
- ☐ American Indian / Alaska Native:
- ☐ Middle Eastern / North African:
- ☐ Native Hawaiian or Other Pacific Islander:
- ☐ Other Not Listed Here, Please Specify

10. There are certain genetic mutations that may lead to GACI or ARHR2. Do you know your/your child's specific mutation?*

- ◯ I or my child HAS NOT BEEN TESTED, I do not know the mutation
- ◯ I or my child HAS BEEN TESTED, but I do not know or remember the mutation
- ◯ Yes, it is noted here

11. If you would like, please note the name and city of the physician who you / your child currently see for GACI or ARHR2, if you choose to do so, we will send them information and a weblink for a free genetic testing program available. We will not share any information from this survey. Your name or information will not be shared in any way.

Please provide First Name, Last Name and City of the physician

Questions 12 - 14 pertain to other members of your immediate and extended family.

12. Please think about your family members, such as aunts, uncles, cousins or siblings:

Note: if not applicable, put "N/A". If, for example you have two persons in your family for whom you are reporting, note them in order -- e.g. "Aunt/GACI, Uncle/ARHR2", and for subsequent rows, report in the same order with your Aunt's information first, and your Uncle's second.

Has anyone in your family (YOUR siblings, parents, cousins, etc.) been diagnosed with GACI or ARHR2 or any form of rickets? If yes, note the diagnosis and relationship. ex Cousin / ARHR2:

Have they undergone genetic testing? (in same order as above):

Do they have a genetic mutation consistent with GACI or ARHR2? (In same order as above. If you do know know, put "unsure"):

If you know their genetic mutation, please note it here: (In same order as above. If you don't know it, put "n/a")

13. Please select the options that will help us understand this family members ethnicity.
Please select all that apply.
    - ☐ White / Caucasian:
    - ☐ Hispanic / Latino or Spanish:
    - ☐ Black / African American:
    - ☐ Asian or Asian Indian:
    - ☐ American Indian / Alaska Native:
    - ☐ Middle Eastern / North African:
    - ☐ Native Hawaiian or Other Pacific Islander:
    - ☐ Other Not Listed Here, Please Specify

14. Again, thinking of your family: Are there any other family members who have experienced any of the following symptoms?

    Issues related to bone weakening (e.g. osteoporosis, stress fractures, or atypical fractures)
    Issues related to bone and joint pain/stiffness
    Issues related to ANY organ calcification
    Issues related to hearing loss
    Issues related to delays in growth (e.g. short stature, delayed developmental milestones)
    Death of child in utero or immediately after birth?

    Please consider symptoms, even if the person has never been suspected of having a disorder related to GACI or ARHR2.

    If the answer is yes, please note the person and the symptoms (e.g. "Aunt who is now deceased had osteoporosis in her 20's. Uncle who is still living has hearing loss ")

RSVP- Understanding the Spectrum of ENPP1 deficiency and ABCC6 deficiency (GACI and ARHR2) Through the Eyes of Patients and Parents

Interview Scheduling

15. Please note a date and time that will work for you to participate in a 40 minute interview between now and July 31, 2020. Please provide a first, second and third choice. We will do everything we can to provide you your first or second choice. Times outside of standard business hours are available, as are weekend hours.*

Please note a date between now and INSERT DATE. Please note a time, specifying AM or PM and your time zone.

	Day of Week (M, T, W, TH, F, SA, SU):	Month (INSERT MONTH):	Date:	Time (hour):	AM or PM:	Time Zone:
First Choice						
Second Choice						
Third Choice						

16. Please select who filled out the Brief Pain Index:

◯ Child between age of 12-18
◯ Parent/Caregiver
◯ Adult Study Participant

17. As a final step, you will now upload any documents you were given links to fill out and submit a proof of disease form. Please note, not all participants will have been directed to the first two forms, as they are based on age.

1) The Patient Reported Quality of Life
2) The Brief Pain Index
3) Proof of disease form. This is **any document that ties your / your child's name to the diagnosis of ENPP1 deficiency, ABCC6 deficiency, GACI or ARHR2**. Examples include a genetic test report, a page from your electronic medical record or others.

Please scan all three documents together, and save, naming the file with your name.

If you are unable to scan the documents or upload the file, you can also email the file to scalton@engagehealth.com or call 1(651)994-0510 for assistance.

Select file to upload:

(click "Browse" button below to locate file)

File size restricted to: 400 KB

File type restricted to: No file type restrictions.

Choose File No file chosen Upload

File Name: (limit 255 characters)

File Description: (limit 255 characters)

Files Uploaded:

You have now completed the survey / RSVP. **Please hit "next" to submit your responses.** Someone from the Engage Health staff will reach out to you to schedule your appointment, or to assist you in providing your files (if they have not been uploaded).

18. For interviewer use only.

RSVP- Understanding the Spectrum of ENPP1 deficiency and ABCC6 deficiency (GACI and ARHR2) Through the Eyes of Patients and Parents

Data Collection Site for Interviewer to Enter Data

GACI / ARHR2 data collection site

This site will be used to collect data for GACI or ARHR2 patient/parent interviews that are conducted by Engage Health and recorded by MP3 file if the patient agrees to be recorded. Engage Health will use this site as the single record for the patient. Information from pages 1 -5 (the welcome, consent, about you, quality of life and interview scheduling) will be completed by the patient/parent and those results will come to Engage Health. Engage Health reviews responses and "proof of disease" and if the respondent qualifies, the interview is scheduled. If the respondent does not qualify, they are notified of that.

At the time of the interview, Engage Health will access the site using a code unique to the patient, and to enter information from the interview in order that the information is tied together from the RSVP and the interview. All information from patient/parent-provided fields on pages 1-5 will be used to pre-populate the fields for the interviewer as the interview is conducted. Pages 6 and greater are used as the interview guide. Please note, this is not a "script", but a guide that captures the questions that the interviewer will ask and the general order in which they will be asked. All answers will be entered by the interviewer into the web-based module.

RSVP- Understanding the Spectrum of ENPP1 deficiency and ABCC6 deficiency (GACI and ARHR2) Through the Eyes of Patients and Parents

Introduction and Authorization to Record & Disease Burden- Unaided

Hello < > this is __ from Engage Health calling for our interview. Does this time still work for you? (If yes, continue)

Engage Health is a health research company that assists clients who are developing therapies for various rare diseases. In this case, we are working with GACI Global and Inozyme Pharma to learn about the burden of GACI or ARHR2 to patients as well as the impact the disease has on their lives.

Inozyme Pharma is a small pharma company developing treatments for rare calcification disorders. GACO Global is the patient advocacy group for people affected by GACI/ARHR2.

We will spend about 40 minutes together, and I envision this being very informal. If I ask something in a way that you are not clear what I'm after, or if you don't believe I've captured your thoughts, please just let me know, OK?

While the research results will be shared with the client, your responses will **not** be tied directly to your name. However to be sure I've captured your thoughts accurately, I'm wondering if I can have your approval to record our discussion, is that OK? After I turn on the tape I will not use your name.

If OK, turn on tape

"Before we begin, I want to be very respectful of your time and, because we have a lot to get through within the time frame for the interview, please forgive me in advance if I have to cut you short or rush things along to ensure we get through everything in this short time that we have together, OK?"

Begin Interview

Before we get into the details related to GACI / ARHR2, I want to make sure I understand a little bit about you and your family **(all < > represent pre-filled fields from the RSVP)**

19. *I understand you are a <> (patient or parent)*
and you / your child was born in <> (year) , diagnosed in <> with <> and is of <> descent.

. . Is that correct?

(Fill in appropriate blanks)

Diagnosis (GACI or ARHR2):	
Yr of Birth:	
Yr of Diagnosis:	

Yr of Passing, if Applicable:

Parent/Caregiver or Patient:

20. You noted that you / your child is of <> descent, is there any other information about your ethnic heritage that you would like to share?

Prefills from Q 9

As you know, this study is about understanding the burdens that you/ your child experience due to GACI or ARHR2.

NOTE TO INTERVIEWER; IF YOU ARE SPEAKING TO THE PARENT OF A CHILD WHO HAS PASSED AWAY, YOU WILL SAY: As you know, this study is about understanding the burdens that are experienced due to GACI or ARHR2. For these questions I would like you to focus on the time before your child's passing. (NOTE TO INTERVIEWER: MOST INFANTS DIE WITHIN FIRST MONTHS)

21. What is/was the ***most important* burden** for you/ your child related to GACI or ARHR2?*

22. Can you tell me more about this? *
Note to interviewer: Attempt to quantify - for example, if they say they can't play sports because of heart issues, ask if they cant play any at all, or if they can have moderate exertion or just have to avoid contract . Try and determine the function on a scale of 1-10 .

23. Under what circumstances does/did it occur?*

24. How often does/did this impact you/your child?
-Never
-Once per year
-Twice to four times per year
-Monthly
-X Times per Month
-Weekly
-X Times per Week
-Daily
-X Times per Day*

25. Why is/was this thing important to you/your child?*

26. How does/did this impact you/your child on a day to day basis?

27. What is/was the ***second most important burden for you/ your child related to GACI or ARHR2?****

28. Can you tell me more about this?*

Note to interviewer: Attempt to quantify - for example, if they say they can't play sports because of heart issues, ask if they cant play any at all, or if they can have moderate exertion or just have to avoid contract . Try and determine the function on a scale of 1-10 .

29. Under what circumstances does/did it occur?*

30. How often does/dd this impact you/your child?

-Never
-Once per year
-Twice to four times per year
-Monthly
-X Times per Month
-Weekly
-X Times per Week
-Daily
-X Times per Day*

31. Why is/was this thing important to you/your child?*

32. How does/did this impact you/your child on on a day to day basis?

33. What is/was the **third** ***most important burden for you/ your child related to GACI or ARHR2?***

*

34. Can you tell me more about this?*

Note to interviewer: Attempt to quantify - for example, if they say they can't play sports because of heart issues, ask if they cant play any at all, or if they can have moderate exertion or just have to avoid contract . Try and determine the function on a scale of 1-10 .

35. Under what circumstances does/did it occur?*

36. How often does/did this impact you/your child?
-Never
-Once per year
-Twice to four times per year
-Monthly
-X Times per Month
-Weekly
-X Times per Week
-Daily
-X Times per Day*

37. Why is/was this thing important to you/your child?*

38. How does/did this impact you/your child on on a day to day basis?

Disease Burden -- Aided

For this part of the interview I am going to ask you to review some categories of things that might be considered "symptoms" of GACI / ARHR2. I am going to ask you to go to your email. I have just sent you a link for a "Zoom meeting". Click on that and let me know when you can see a document.

Can you see a document?
(Note to interviewer; if you can't get it to work, email the word document "Interview Question Categories") The participant will see the list in Q39 as a WORD document. They will NOT see any of the questions or answer boxes that the interviewer fills in).

In this section, I want you to review this list of things and tell me ALL of the issues listed that you / your child currently experiences or has experienced in the past. *(Note to interviewer;if the child has passed away, you will say "In this section, I want you to review this list of things and tell me ALL of the issues listed that your child experienced when he/she was living).*

After you review the list, we will talk about which of these issues is/was the most burdensome to you/your child ; if none of these would describe this, tell me what would

39. Select ALL symptoms that you currently experience or have experienced in the past:
OR
Select ALL of the symptoms that your child experienced when he/she was living

NOTE TO INTERVIEWER, GO THROUGH THE LIST AND ASK ABOUT EACH. WHEN THROUGH THE LIST, ASK "Are there others that are not listed here that I should add? If so, what are they? KEEP GOING UNTIL YOU HAVE EXHAUSTED ALL SYMPTOMS, EVEN IF THEY ARE NOT SURE THEY ARE RELATED TO GACI OR ARHR2*

- ☐ Physical Health- related to heart issues
- ☐ Physical Health- related to gastrointestinal (stomach) issues
- ☐ Physical Health- related to bone / joint pain
- ☐ Physical Health- related to joint stiffness
- ☐ Physical Health- related to mobility and fatigue
- ☐ Physical Health- related to growth and development such as feeding issues, failure to thrive (slow growth) short stature and developmental delays
- ☐ Physical Health- related to hearing loss
- ☐ Physical Health- related to renal (kidney) impairment
- ☐ Social Health- peer relationships
- ☐ Social Health- family relationships
- ☐ Mental Health- related to fear of unknown
- ☐ Mental Health- related to stress / anxiety
- ☐ Other, please specify all others

____

RSVP- Understanding the Spectrum of ENPP1 deficiency and ABCC6 deficiency (GACI and ARHR2) Through the Eyes of Patients and Parents

Follow Up Questions from Q40

40. You noted Physical Health- related to **heart issues**. What specific symptoms occurred? When did they occur? At what age did these events occur?

Symptoms that occurred:

When they occurred:

Age at which they occurred:

41. You noted Physical Health- related to **heart issues**. Have you had any interventions, i.e. valve replacement, heart transplant, etc.

Please list all interventions they say.

42. You noted, Physical Health- related to **heart issues**. Have these symptoms resolved, gotten worse, or stayed the same?

- Symptom Stayed the Same
- Symptom Gotten Worse
- Symptom Resolved
- Other, please specify

43. You noted Physical Health- related to **gastrointestinal (stomach issues)**. What specific symptoms occurred? When did they occur? At what age did these events occur?

Symptoms that occurred:

When they occurred:

Age at which they occurred:

44. You noted Physical Health- related to **gastrointestinal (stomach issues)**. Have you had any interventions, i.e.

Please list all interventions they say.

45. You noted, Physical Health- related to **gastrointestinal (stomach issues)**. Have these symptoms resolved, gotten worse, or stayed the same?

- Symptom Stayed the Same
- Symptom Gotten Worse
- Symptom Resolved
- Other, please specify

46. You noted Physical Health- related to **bone / joint pain**. What specific symptoms occurred? When did they occur? At what age did these events occur?

Symptoms that occurred:

When they occurred:

Age at which they occurred:

47. You noted Physical Health- related to **bone / joint pain**. Was surgical intervention required? If surgical intervention was required. When did it occur?

Please list all interventions they say.

48. You noted that you have experienced Physical Health- related to **bone / joint pain**. Have you had any of the following?

Please check all the issues they have had.

- ☐ Fractures
- ☐ Rickets
- ☐ Calcification in Joints
- ☐ Ensopathies (issues with ligament or tendon attachment)
- ☐ Other, please specify

49. You noted, Physical Health- related to **bone / joint pain**. Have these symptoms resolved, gotten worse, or stayed the same?

- ○ Symptom Stayed the Same
- ○ Symptom Gotten Worse
- ○ Symptom Resolved
- ○ Other, please specify

50. You noted Physical Health- related to **joint stiffness**. What specific symptoms occurred? When did they occur? At what age did these events occur?

Symptoms that occurred:

When they occurred:

Age at which they occurred:

51. You noted Physical Health- related to **joint stiffness**. Have you had any interventions, i.e.

Please list all interventions they say.

52. You noted Physical Health- related to **joint stiffness**. Is the joint stiffness in the Upper Extremity or Lower Extremity?

- ☐ Upper Extremity
- ☐ Lower Extremity

53. You noted Physical Health- related to **joint stiffness**. On a scale of 1 - 10 with 1 being "no pain" 10 being "excruciating pain". How much pain have you experienced related to joint stiffness within the last 30 days.

	1	2	3	4	5	6	7	8	9	10	
1 No Pain	○	○	○	○	○	○	○	○	○	○	10 Excruciating Pain

54. You noted, Physical Health- related to **joint stiffness**. Have these symptoms resolved, gotten worse, or stayed the same?

- Symptom Stayed the Same
- Symptom Gotten Worse
- Symptom Resolved
- Other, please specify

55. You noted Physical Health- related to **mobility and fatigue**. What specific symptoms occurred? When did they occur? At what age did these events occur?

Symptoms that occurred:

When they occurred:

Age at which they occurred:

56. You noted Physical Health- related to **mobility and fatigue**. Have you had any interventions, i.e.

Please list all interventions they say.

57. You noted Physical Health- related to **mobility and fatigue**. Do you experience any fatigue?

If fatigue, prompt if they are tired at rest or fatigued only if they perform an activity such as walking, running, etc.

	Yes/No	When do you feel fatigued?
Fatigue?	-- Please Sel	-- Please Sel

58. You noted Physical Health- related to **mobility and fatigue**. What type of assistive devices are used due to mobility?

Please check all that they say

- Cane/Walker
- Wheelchair
- Scooter
- Grabber
- Other, please specify

59. You noted, Physical Health- related to **mobility and fatigue**. Have these symptoms resolved, gotten worse, or stayed the same?

- Symptom Stayed the Same
- Symptom Gotten Worse
- Symptom Resolved
- Other, please specify

60. You noted Physical Health- related to **growth and development such as feeding issues, failure to thrive (slow growth) short stature and developmental delays**. What specific symptoms occurred? When did they occur? At what age did these events occur?

Symptoms that occurred:

When they occurred:

Age at which they occurred:

61. You noted Physical Health- related to **growth and development such as feeding issues, failure to thrive (slow growth) short stature and developmental delays**. Have you had any interventions, i.e.

Please list all interventions they say.

62. You noted, Physical Health- related to **growth and development such as feeding issues, failure to thrive (slow growth) short stature and developmental delays**. Have these symptoms resolved, gotten worse, or stayed the same?

- Symptom Stayed the Same
- Symptom Gotten Worse
- Symptom Resolved
- Other, please specify

63. You noted Physical Health- related to **hearing loss**. What specific symptoms occurred? When did they occur? At what age did these events occur?

Symptoms that occurred:

When they occurred:

Age at which they occurred:

64. You noted Physical Health- related to **hearing loss**. Have you had any interventions, i.e.

Please list all interventions they say.

65. You noted Physical Health- related to **hearing loss**. What type of issues / devices have you had in relation to hearing loss.

Please check all that they say

- Hearing Aid
- Hearing Loss
- Cochlear implants
- Other, please specify

66. You noted Physical Health- related to **hearing loss**. What degree of hearing loss do you have?

- Normal
- Slight
- Mild
- Moderate
- Moderately Severe
- Severe
- Profound

67. You noted Physical Health- related to **hearing loss**. At what age did these issues onset?

68. You noted, Physical Health- related to **hearing loss**. Have these symptoms resolved, gotten worse, or stayed the same?

- Symptom Stayed the Same
- Symptom Gotten Worse
- Symptom Resolved
- Other, please specify

69. You noted Physical Health- related to **renal (kidney) impairment**. What specific symptoms occurred? When did they occur? At what age did these events occur?

Symptoms that occurred:

When they occurred:

Age at which they occurred:

70. You noted Physical Health- related to **renal (kidney) impairment**. Have you had any interventions, i.e.
Please list all interventions they say.

71. You noted Physical Health- related to **renal (kidney) impairment**. Have you had any of the following issues.
Please check all that they say

- ☐ Nephrocalcinosis (high kidney calcium levels)
- ☐ Renal/kidney failure
- ☐ Problems with phosphate wasting
- ☐ Other, please specify

72. You noted, Physical Health- related to **renal (kidney) impairment**. Have these symptoms resolved, gotten worse, or stayed the same?

- ◯ Symptom Stayed the Same
- ◯ Symptom Gotten Worse
- ◯ Symptom Resolved
- ◯ Other, please specify

73. You noted Social Health- **peer relationships**. What specific symptoms occurred? When did they occur? At what age did these events occur?

Symptoms that occurred:

When they occurred:

Age at which they occurred:

74. You noted Social Health- **peer relationships**. Have you had any interventions, i.e.
Please list all interventions they say.

75. You noted, Social Health- **peer relationships**. Have these symptoms resolved, gotten worse, or stayed the same?

- ◯ Symptom Stayed the Same
- ◯ Symptom Gotten Worse
- ◯ Symptom Resolved
- ◯ Other, please specify

76. You noted Social Health- **family relationships**. What specific symptoms occurred? When did they occur? At what age did these events occur?

Symptoms that occurred:

When they occurred:

Age at which they occurred:

77. You noted Social Health- **family relationships**. Have you had any interventions, i.e.
Please list all interventions they say.

78. You noted, Social Health- **family relationships**. Have these symptoms resolved, gotten worse, or stayed the same?

- Symptom Stayed the Same
- Symptom Gotten Worse
- Symptom Resolved
- Other, please specify

79. You noted Mental Health- related to **fear of unknown**. When did this present, and at what age did this occur?

Symptoms that occurred:

When they occurred:

Age at which they occurred:

80. You noted Mental Health- related to **fear of unknown**. Have you had any interventions, i.e.

Please list all interventions they say.

81. You noted, Mental Health- related to **fear of unknown**. Have these symptoms resolved, gotten worse, or stayed the same?

- Symptom Stayed the Same
- Symptom Gotten Worse
- Symptom Resolved
- Other, please specify

82. You noted Mental Health- related to **stress / anxiety**. What specific symptoms occurred? When did they occur? At what age did these events occur?

Symptoms that occurred:

When they occurred:

Age at which they occurred:

83. You noted Mental Health- related to **stress / anxiety**. Have you had any interventions, i.e.

Please list all interventions they say.

84. You noted, Mental Health- related to **stress / anxiety**. Have these symptoms resolved, gotten worse, or stayed the same?

- Symptom Stayed the Same
- Symptom Gotten Worse
- Symptom Resolved
- Other, please specify

85. You noted **Other**. What specific symptoms occurred? When did they occur? At what age did these events occur?

NOTE TO INTERVIEWER: ONLY FILL IF OTHER WAS SELECTED

Symptoms that occurred: ____________________

When they occurred: ____________________

Age at which they occurred: ____________________

86. You noted **Other**. Have you had any interventions.

NOTE TO INTERVIEWER: ONLY FILL IF OTHER WAS SELECTED

Please list all interventions they say.

____

87. You noted, **Other**. Have these symptoms resolved, gotten worse, or stayed the same?

NOTE TO INTERVIEWER: ONLY FILL IF OTHER WAS SELECTED

- ○ Symptom Stayed the Same
- ○ Symptom Gotten Worse
- ○ Symptom Resolved
- ○ Other, please specify ____________________

RSVP- Understanding the Spectrum of ENPP1 deficiency and ABCC6 deficiency (GACI and ARHR2) Through the Eyes of Patients and Parents

Categories of Things That Burden Me/My Child

88. Next, we are going to talk about which of these are/were the MOST IMPORTANT or MOST IMPACTFUL to you / your child.*

- ◯ Physical Health- related to heart issues
- ◯ Physical Health- related to gastrointestinal (stomach) issues
- ◯ Physical Health- related to bone / joint pain
- ◯ Physical Health- related to joint stiffness
- ◯ Physical Health- related to mobility and fatigue
- ◯ Physical Health- related to growth and development such as feeding issues, failure to thrive (slow growth) short stature and developmental delays
- ◯ Physical Health- related to hearing loss
- ◯ Physical Health- related to renal (kidney) impairment
- ◯ Social Health- peer relationships
- ◯ Social Health- family relationships
- ◯ Mental Health- related to fear of unknown
- ◯ Mental Health- related to stress / anxiety
- ◯ Other, please specify all others

____

RSVP- Understanding the Spectrum of ENPP1 deficiency and ABCC6 deficiency (GACI and ARHR2) Through the Eyes of Patients and Parents

Categories of Things That Burden Me/My Child

89. What *specifically* from Physical Health- related to **heart issues** is it that burdens/burdened you/your child because of this disease?*

90. What *specifically* does/did Physical Health- related to **heart issues** prevent you/your child from doing, that you/they wish they could?*

91. What *specifically* from Physical Health- related to **gastrointestinal (stomach) issues** is it that burdens/burdened you/your child because of this disease?*

92. What *specifically* does/did Physical Health- related to **gastrointestinal (stomach) issues** prevent you/your child from doing, that you/they wish they could? *

93. What *specifically* from Physical Health- related to **bone / joint pain** is it that burdens/burdened you/your child because of this disease?*

94. What *specifically* does/did Physical Health- related to **bone / joint pain** prevent you/your child from doing, that you/they wish they could? *

95. What *specifically* from Physical Health- related to **joint stiffness** is it that burdens/burdened you/your child because of this disease?*

96. What *specifically* does/did Physical Health- related to **joint stiffness** prevent you/your child from doing, that you/they wish they could? *

97. What *specifically* from Physical Health- related to **mobility and fatigue** is it that burdens/burdened you/your child because of this disease?*

98. What *specifically* does/did Physical Health- related to **mobility and fatigue** prevent you/your child from doing, that you/they wish they could? *

99. What *specifically* from Physical Health- related to **growth and development such as feeding issues, failure to thrive (slow growth) short stature and developmental delays** is it that burdens/burdened you/your child because of this disease?*

100. What *specifically* does/did Physical Health- related to **growth and development such as feeding issues, failure to thrive (slow growth) short stature and developmental delays** prevent you/your child from doing, that you/they wish they could? *

101. What *specifically* from Physical Health- related to **hearing loss** is it that burdens/burdened you/your child because of this disease?*

102. What *specifically* does/did Physical Health- related to **hearing loss** prevent you/your child from doing, that you/they wish they could? *

103. What *specifically* from Physical Health- related to **renal (kidney) impairment** is it that burdens/burdened you/your child because of this disease?*

104. What *specifically* does/did Physical Health- related to **renal (kidney) impairment** prevent you/your child from doing, that you/they wish they could?*

105. What *specifically* from **Social Health peer relationships** is it that burdens/burdened you/your child because of this disease?*

106. What *specifically* does/did **Social Health peer relationships** prevent you/your child from doing, that you/they wish they could? *

107. What *specifically* from **Social Health family relationships** is it that burdens/burdened you/your child because of this disease?*

108. What *specifically* does/did **Social Health family relationships** prevent you/your child from doing, that you/they wish they could? *

109. What *specifically* from **Mental Health related to fear of unknown** is it that burdens/burdened you/your child because of this disease?*

110. What *specifically* does/did **Mental Health related to fear of unknown** prevent you/your child from doing, that you/they wish they could? *

111. What *specifically* from **Mental Health related to stress / anxiety** is it that burdens/burdened you/your child because of this disease?*

112. What *specifically* does/did **Mental Health related to stress / anxiety** prevent you/your child from doing, that you/they wish they could? *

113. What *specifically* from ______ is it that burdens/burdened you/your child because of this disease?

NOTE TO INTERVIEWER: ONLY FILL IF OTHER WAS SELECTED

Interviewer; note "other" here before response

114. What *specifically* does/did ________ prevent you/your child from doing, that you/they wish they could?

NOTE TO INTERVIEWER: ONLY FILL IF OTHER WAS SELECTED

Interviewer, note "other" here before response

115. How often does/did this impact you/your child?

-Never
-Once per year
-Twice to four times per year
-Monthly
-X Times per Month
-Weekly
-X Times per Week
-Daily
-X Times per Day

116. Why is/was this thing important to you/your child?

117. How does/did this impact you/your child on a day to day basis?

RSVP- Understanding the Spectrum of ENPP1 deficiency and ABCC6 deficiency (GACI and ARHR2) Through the Eyes of Patients and Parents

Life Impact

At this point, the interviewer shows the list of life impacts shown in Q 60 which the participant sees as a second WORD document.

118. When there are things that are burdens, they impact different people in different ways. For example, if we both can't travel that may matter to you because of reasons related to social issues / connections with others, but for me it matters because it limits my job choices and hurts my financial situation. You mentioned that **Physical Health- related to heart issues** was most important to you/your child - Please review the list on my screen and tell me what part of your/your child's life does that impact the most?*

- ◯ Self-esteem/Self-confidence: Being comfortable with others
- ◯ Connection with others: Being able to form meaningful peer relationships, being less lonely, being accepted
- ◯ Financial situation: Having money to pay for things, have to pay for so many medications/equipment out of pocket
- ◯ Time commitment: Spend a lot of time talking to my insurance company, spend a lot of time talking to doctors/healthcare providers, spend a lot of time figuring out how to get places or how to do things that are hard for me, spend a lot of time dealing with caretakers
- ◯ Independence: The ability to do things on own, live on own, not be dependent on others
- ◯ Inclusion: Being included in extracurricular activities/recreation/ sports, being part of a group
- ◯ Other, please specify

119. When there are things that are burdens, they impact different people in different ways. For example, if we both can't travel that may matter to you because of reasons related to social issues / connections with others, but for me it matters because it limits my job choices and hurts my financial situation. You mentioned that **Physical Health- related to gastrointestinal (stomach) issues** was most important to you/your child - Please review the list on my screen and tell me what part of your/your child's life does that impact the most?*

- ◯ Self-esteem/Self-confidence: Being comfortable with others
- ◯ Connection with others: Being able to form meaningful peer relationships, being less lonely, being accepted
- ◯ Financial situation: Having money to pay for things, have to pay for so many medications/equipment out of pocket
- ◯ Time commitment: Spend a lot of time talking to my insurance company, spend a lot of time talking to doctors/healthcare providers, spend a lot of time figuring out how to get places or how to do things that are hard for me, spend a lot of time dealing with caretakers
- ◯ Independence: The ability to do things on own, live on own, not be dependent on others
- ◯ Inclusion: Being included in extracurricular activities/recreation/ sports, being part of a group
- ◯ Other, please specify

120. When there are things that are burdens, they impact different people in different ways. For example, if we both can't travel that may matter to you because of reasons related to social issues / connections with others, but for me it matters because it limits my job choices and hurts my financial situation. You mentioned that **Physical Health- related to bone / joint pain** was most important to you/your child - Please review the list on my screen and tell me what part of your/your child's life does that impact the most?*

- ◯ Self-esteem/Self-confidence: Being comfortable with others
- ◯ Connection with others: Being able to form meaningful peer relationships, being less lonely, being accepted
- ◯ Financial situation: Having money to pay for things, have to pay for so many medications/equipment out of pocket

- Time commitment: Spend a lot of time talking to my insurance company, spend a lot of time talking to doctors/healthcare providers, spend a lot of time figuring out how to get places or how to do things that are hard for me, spend a lot of time dealing with caretakers
- Independence: The ability to do things on own, live on own, not be dependent on others
- Inclusion: Being included in extracurricular activities/recreation/ sports, being part of a group
- Other, please specify

121. When there are things that are burdens, they impact different people in different ways. For example, if we both can't travel that may matter to you because of reasons related to social issues / connections with others, but for me it matters because it limits my job choices and hurts my financial situation. You mentioned that **Physical Health- related to joint stiffness** was most important to you/your child - Please review the list on my screen and tell me what part of your/your child's life does that impact the most?*
    - Self-esteem/Self-confidence: Being comfortable with others
    - Connection with others: Being able to form meaningful peer relationships, being less lonely, being accepted
    - Financial situation: Having money to pay for things, have to pay for so many medications/equipment out of pocket
    - Time commitment: Spend a lot of time talking to my insurance company, spend a lot of time talking to doctors/healthcare providers, spend a lot of time figuring out how to get places or how to do things that are hard for me, spend a lot of time dealing with caretakers
    - Independence: The ability to do things on own, live on own, not be dependent on others
    - Inclusion: Being included in extracurricular activities/recreation/ sports, being part of a group
    - Other, please specify

122. When there are things that are burdens, they impact different people in different ways. For example, if we both can't travel that may matter to you because of reasons related to social issues / connections with others, but for me it matters because it limits my job choices and hurts my financial situation. You mentioned that **Physical Health- related to mobility and fatigue** was most important to you/your child - Please review the list on my screen and tell me what part of your/your child's life does that impact the most?*
    - Self-esteem/Self-confidence: Being comfortable with others
    - Connection with others: Being able to form meaningful peer relationships, being less lonely, being accepted
    - Financial situation: Having money to pay for things, have to pay for so many medications/equipment out of pocket
    - Time commitment: Spend a lot of time talking to my insurance company, spend a lot of time talking to doctors/healthcare providers, spend a lot of time figuring out how to get places or how to do things that are hard for me, spend a lot of time dealing with caretakers
    - Independence: The ability to do things on own, live on own, not be dependent on others
    - Inclusion: Being included in extracurricular activities/recreation/ sports, being part of a group
    - Other, please specify

123. When there are things that are burdens, they impact different people in different ways. For example, if we both can't travel that may matter to you because of reasons related to social issues / connections with others, but for me it matters because it limits my job choices and hurts my financial situation. You mentioned that **Physical Health- related to growth and development such as feeding issues, failure to thrive (slow growth) short stature and developmental delays** was most important to you/your child - Please review the list on my screen and tell me what part of your/your child's life does that impact the most?*
    - Self-esteem/Self-confidence: Being comfortable with others
    - Connection with others: Being able to form meaningful peer relationships, being less lonely, being accepted
    - Financial situation: Having money to pay for things, have to pay for so many medications/equipment out of pocket
    - Time commitment: Spend a lot of time talking to my insurance company, spend a lot of time talking to doctors/healthcare providers, spend a lot of time figuring out how to get places or how to do things that are hard for me, spend a lot of time dealing with caretakers
    - Independence: The ability to do things on own, live on own, not be dependent on others
    - Inclusion: Being included in extracurricular activities/recreation/ sports, being part of a group
    - Other, please specify

124. When there are things that are burdens, they impact different people in different ways. For example, if we both

can't travel that may matter to you because of reasons related to social issues / connections with others, but for me it matters because it limits my job choices and hurts my financial situation. You mentioned that **Physical Health- related to hearing loss** was most important to you/your child - Please review the list on my screen and tell me what part of your/your child's life does that impact the most?*

- Self-esteem/Self-confidence: Being comfortable with others
- Connection with others: Being able to form meaningful peer relationships, being less lonely, being accepted
- Financial situation: Having money to pay for things, have to pay for so many medications/equipment out of pocket
- Time commitment: Spend a lot of time talking to my insurance company, spend a lot of time talking to doctors/healthcare providers, spend a lot of time figuring out how to get places or how to do things that are hard for me, spend a lot of time dealing with caretakers
- Independence: The ability to do things on own, live on own, not be dependent on others
- Inclusion: Being included in extracurricular activities/recreation/ sports, being part of a group
- Other, please specify

125. When there are things that are burdens, they impact different people in different ways. For example, if we both can't travel that may matter to you because of reasons related to social issues / connections with others, but for me it matters because it limits my job choices and hurts my financial situation. You mentioned that **Physical Health- related to renal (kidney) impairment** was most important to you/your child - Please review the list on my screen and tell me what part of your/your child's life does that impact the most?*

- Self-esteem/Self-confidence: Being comfortable with others
- Connection with others: Being able to form meaningful peer relationships, being less lonely, being accepted
- Financial situation: Having money to pay for things, have to pay for so many medications/equipment out of pocket
- Time commitment: Spend a lot of time talking to my insurance company, spend a lot of time talking to doctors/healthcare providers, spend a lot of time figuring out how to get places or how to do things that are hard for me, spend a lot of time dealing with caretakers
- Independence: The ability to do things on own, live on own, not be dependent on others
- Inclusion: Being included in extracurricular activities/recreation/ sports, being part of a group
- Other, please specify

126. When there are things that are burdens, they impact different people in different ways. For example, if we both can't travel that may matter to you because of reasons related to social issues / connections with others, but for me it matters because it limits my job choices and hurts my financial situation. You mentioned that **Social Health- peer relationships** was most important to you/your child - Please review the list on my screen and tell me what part of your/your child's life does that impact the most?*

- Self-esteem/Self-confidence: Being comfortable with others
- Connection with others: Being able to form meaningful peer relationships, being less lonely, being accepted
- Financial situation: Having money to pay for things, have to pay for so many medications/equipment out of pocket
- Time commitment: Spend a lot of time talking to my insurance company, spend a lot of time talking to doctors/healthcare providers, spend a lot of time figuring out how to get places or how to do things that are hard for me, spend a lot of time dealing with caretakers
- Independence: The ability to do things on own, live on own, not be dependent on others
- Inclusion: Being included in extracurricular activities/recreation/ sports, being part of a group
- Other, please specify

127. When there are things that are burdens, they impact different people in different ways. For example, if we both can't travel that may matter to you because of reasons related to social issues / connections with others, but for me it matters because it limits my job choices and hurts my financial situation. You mentioned that **Social Health- family relationships** was most important to you/your child - Please review the list on my screen and tell me what part of your/your child's life does that impact the most?*

- Self-esteem/Self-confidence: Being comfortable with others
- Connection with others: Being able to form meaningful peer relationships, being less lonely, being accepted
- Financial situation: Having money to pay for things, have to pay for so many medications/equipment out of pocket
- Time commitment: Spend a lot of time talking to my insurance company, spend a lot of time talking to doctors/healthcare providers, spend a lot of time figuring out how to get places or how to do things that are hard for me, spend a lot of time dealing with caretakers

- Independence: The ability to do things on own, live on own, not be dependent on others
- Inclusion: Being included in extracurricular activities/recreation/ sports, being part of a group
- Other, please specify

128. When there are things that are burdens, they impact different people in different ways. For example, if we both can't travel that may matter to you because of reasons related to social issues / connections with others, but for me it matters because it limits my job choices and hurts my financial situation. You mentioned that **Mental Health- related to fear of unknown** was most important to you/your child - Please review the list on my screen and tell me what part of your/your child's life does that impact the most?*
    - Self-esteem/Self-confidence: Being comfortable with others
    - Connection with others: Being able to form meaningful peer relationships, being less lonely, being accepted
    - Financial situation: Having money to pay for things, have to pay for so many medications/equipment out of pocket
    - Time commitment: Spend a lot of time talking to my insurance company, spend a lot of time talking to doctors/healthcare providers, spend a lot of time figuring out how to get places or how to do things that are hard for me, spend a lot of time dealing with caretakers
    - Independence: The ability to do things on own, live on own, not be dependent on others
    - Inclusion: Being included in extracurricular activities/recreation/ sports, being part of a group
    - Other, please specify

129. When there are things that are burdens, they impact they impact different people in different ways. For example, if we both can't travel that may matter to you because of reasons related to social issues / connections with others, but for me it matters because it limits my job choices and hurts my financial situation. You mentioned that **Mental Health- related to stress / anxiety** was most important to you/your child - Please review the list on my screen and tell me what part of your/your child's life does that impact the most?*
    - Self-esteem/Self-confidence: Being comfortable with others
    - Connection with others: Being able to form meaningful peer relationships, being less lonely, being accepted
    - Financial situation: Having money to pay for things, have to pay for so many medications/equipment out of pocket
    - Time commitment: Spend a lot of time talking to my insurance company, spend a lot of time talking to doctors/healthcare providers, spend a lot of time figuring out how to get places or how to do things that are hard for me, spend a lot of time dealing with caretakers
    - Independence: The ability to do things on own, live on own, not be dependent on others
    - Inclusion: Being included in extracurricular activities/recreation/ sports, being part of a group
    - Other, please specify

130. When there are things that are burdens, they impact different people in different ways. For example, if we both can't travel that may matter to you because of reasons related to social issues / connections with others, but for me it matters because it limits my job choices and hurts my financial situation. You mentioned that **Other** was most important to you/your child - Please review the list on my screen and tell me what part of your/your child's life does that impact the most?

    NOTE TO INTERVIEWER: ONLY FILL IF OTHER WAS SELECTED
    - Self-esteem/Self-confidence: Being comfortable with others
    - Connection with others: Being able to form meaningful peer relationships, being less lonely, being accepted
    - Financial situation: Having money to pay for things, have to pay for so many medications/equipment out of pocket
    - Time commitment: Spend a lot of time talking to my insurance company, spend a lot of time talking to doctors/healthcare providers, spend a lot of time figuring out how to get places or how to do things that are hard for me, spend a lot of time dealing with caretakers
    - Independence: The ability to do things on own, live on own, not be dependent on others
    - Inclusion: Being included in extracurricular activities/recreation/ sports, being part of a group
    - Other, please specify

131. Please share specifically what about this reason makes it/made it important for you/your child:

RSVP- Understanding the Spectrum of ENPP1 deficiency and ABCC6 deficiency (GACI and ARHR2) Through the Eyes of Patients and Parents

Drug Development / Approval Insights

132. Sometimes when drugs are approved by FDA, the "endpoints" or measurements for them to consider effectiveness are sometimes not that meaningful to patients / families.
In order of importance, what are the top three things a drug or therapy should **do** that would be important to you/your child.

*

(Note to interviewer: probe deep - for example, "it should be effective" - figure out what is the thing they want it to do from a functional or physical standpoint, i.e. allow me to control my blood pressure)

1:

2:

3:

133. As we are coming to the end of our time, is there anything else you/your child would like to add that you would like the people the developing or approving new medicines to understand.

134. Can we contact you/your child for clarification, participation in the future, and other study-related updates?*

◯ Yes

◯ No

GACI Global
circulating hope

inozyme pharma

RSVP- Understanding the Spectrum of ENPP1 deficiency and ABCC6 deficiency (GACI and ARHR2) Through the Eyes of Patients and Parents

Thank You

135. We might like to reach out to healthcare providers about future trials that may be of interest. If we reach out to yours, is it okay if we use your/your child's name?*

◯ Yes, I grant this permission
◯ No, I do not grant this permission

136. *May we contact you for follow up or to participate in similar studies?* *

◯ Yes
◯ No

Turn off tape and dismiss client. Verify honoraria type and delivery email / address.

In English: If the respondent has allowed recording, post tape to P Drive

In German or French: If the respondent has allowed recording, send ***using encrypted email*** *to scalton@engagehealth.com May require condensing the file.*

137. As you know you will receive $100 for participating in the survey and interview. You have a choice of receiving a check in the mail (cut in US dollars from a US bank) or an "e-gift card" from Amazon.com. Which do you prefer?

As soon as we finish here this will be processed.*

◯ Check in US $ from US Bank
◯ e-Gift Card from Amazon.com

138. You provided the email <> , is this where you would like your gift card sent? *

◯ Yes
◯ No, the correct email for gift card is noted here: ______

139. Just to confirm this is the name and address you want us to send your check....
Pre-fill from Q 4
*

Name:	
Street Address (where to send your honoraria check):	
City:	
State or Province: (e.g. NY):	
Zip or Postal Code:	
Country:	

140. Check here to notify Engage Scheduling that a check needs to be cut.

○ Check here

RSVP- Understanding the Spectrum of ENPP1 deficiency and ABCC6 deficiency (GACI and ARHR2) Through the Eyes of Patients and Parents

Thank You

Thank you for your interest in the study: Understanding GACI and ARHR2 Through the Eyes of Patients and Parents. Please click "done" to capture your responses. If you are eligible to participate, you will be contacted shortly to confirm your interview or to notify you that interviews are full, if that is the case.

Thank you!

Christine O'Brien
Co-President
GACI Global

Pedro Huertas, MD, PhD
Chief Medical Officer
Inozyme Pharma
